# Mitochondrial remodelling is essential for female germ cell differentiation and survival

**DOI:** 10.1371/journal.pgen.1010610

**Published:** 2023-01-25

**Authors:** Vernon Leander Monteiro, Darya Safavian, Deepika Vasudevan, Thomas Ryan Hurd

**Affiliations:** 1 Department of Molecular Genetics, University of Toronto, Ontario, Canada; 2 Department of Cell Biology, University of Pittsburgh School of Medicine, Pittsburgh Pennsylvania, United States of America; University of Cologne, GERMANY

## Abstract

Stem cells often possess immature mitochondria with few inner membrane invaginations, which increase as stem cells differentiate. Despite this being a conserved feature across many stem cell types in numerous organisms, how and why mitochondria undergo such remodelling during stem cell differentiation has remained unclear. Here, using Drosophila germline stem cells (GSCs), we show that Complex V drives mitochondrial remodelling during the early stages of GSC differentiation, prior to terminal differentiation. This endows germline mitochondria with the capacity to generate large amounts of ATP required for later egg growth and development. Interestingly, impairing mitochondrial remodelling prior to terminal differentiation results in endoplasmic reticulum (ER) lipid bilayer stress, Protein kinase R-like ER kinase (PERK)-mediated activation of the Integrated Stress Response (ISR) and germ cell death. Taken together, our data suggest that mitochondrial remodelling is an essential and tightly integrated aspect of stem cell differentiation. This work sheds light on the potential impact of mitochondrial dysfunction on stem and germ cell function, highlighting ER lipid bilayer stress as a potential major driver of phenotypes caused by mitochondrial dysfunction.

## Author summary

Stem cells are necessary for both tissue development and regeneration. As stem cells differentiate into specialized cells their mitochondria often undergo changes, including the remodelling of their inner mitochondrial membranes. Using a population of adult germline stem cells in the Drosophila ovary, we show that mitochondrial remodelling during differentiation acts to endow germline mitochondria with the capacity to generate large amounts of ATP, which are required for later egg growth and development. Interestingly, we also find that impairing mitochondrial remodelling induces endoplasmic reticulum stress, which results in premature termination of differentiation and eventual cell death. Our work illuminates the potential impact of mitochondrial dysfunction on stem cell function and differentiation, and potential mechanisms of mitochondrial disease.

## Introduction

Mitochondria are essential organelles which perform many critical metabolic functions, most notably the production of ATP via the process of oxidative phosphorylation. Mitochondria also have numerous functions beyond energy metabolism including acting as intracellular signaling platforms that play key roles in cell fate decisions [1–3]. While mitochondria have been studied extensively in isolation and in postmitotic cells, how mitochondria function in stem and progenitor cells to influence cell fate decisions remains much less well understood.

Pluripotent stem cells generally do not rely on mitochondria for their ATP production [2,4]. Yet numerous studies indicate that mitochondrial function is critical for the maintenance of stem cell pluripotency and the initiation of differentiation [3,5–7]. Indeed, recent work suggests a model wherein mitochondrial function plays a direct role in adult stem cell fate and tissue homeostasis [8–11]. However, despite the critical role that ovarian stem and progenitor cells play in female fertility and reproduction, our understanding of the importance of mitochondrial function in female GSCs and their immediate progeny remains very limited.

To identify intrinsic factors required for female GSC function and fertility, we previously conducted a transcriptome-wide screen [12] and discovered an unexpected role for Complex V in female GSC differentiation. Complex V, also known as the mitochondrial ATP synthase, is a 15-subunit complex present in the inner mitochondrial membrane that is essential for both ATP production (via oxidative phosphorylation) and for forming the folds in the inner mitochondrial membrane known as cristae [13–15]. Surprisingly, we found that Complex V is required for GSC differentiation independent of its role in ATP synthesis [12] suggesting a crucial role for cristae formation (hereinafter referred to as mitochondrial remodelling) during GSC differentiation.

A defining, conserved feature of stem cell mitochondria is that they have underdeveloped cristae, which mature and increase in number as stem cells differentiate [16]. However, why mitochondria undergo such remodelling and why mitochondrial remodelling is so critical for the differentiation process itself has remained unknown. Here, we find that Drosophila female germ cells undergo a metabolic rewiring, becoming more dependent on oxidative phosphorylation during the later stages of oogenesis. Despite this, mitochondrial remodelling is still essential for the early stages of differentiation. We find that inhibiting mitochondrial remodelling at these stages causes ER lipid bilayer stress, PERK-mediated activation of the Integrated Stress Response (ISR), which in turn induces premature meiosis, germ cell death and sterility. Our data indicate that mitochondrial remodelling is a critical and integral component of the GSC differentiation program in Drosophila.

## Results

### Mitochondrial remodelling is essential for germline stem cell differentiation

**Fig 1:**
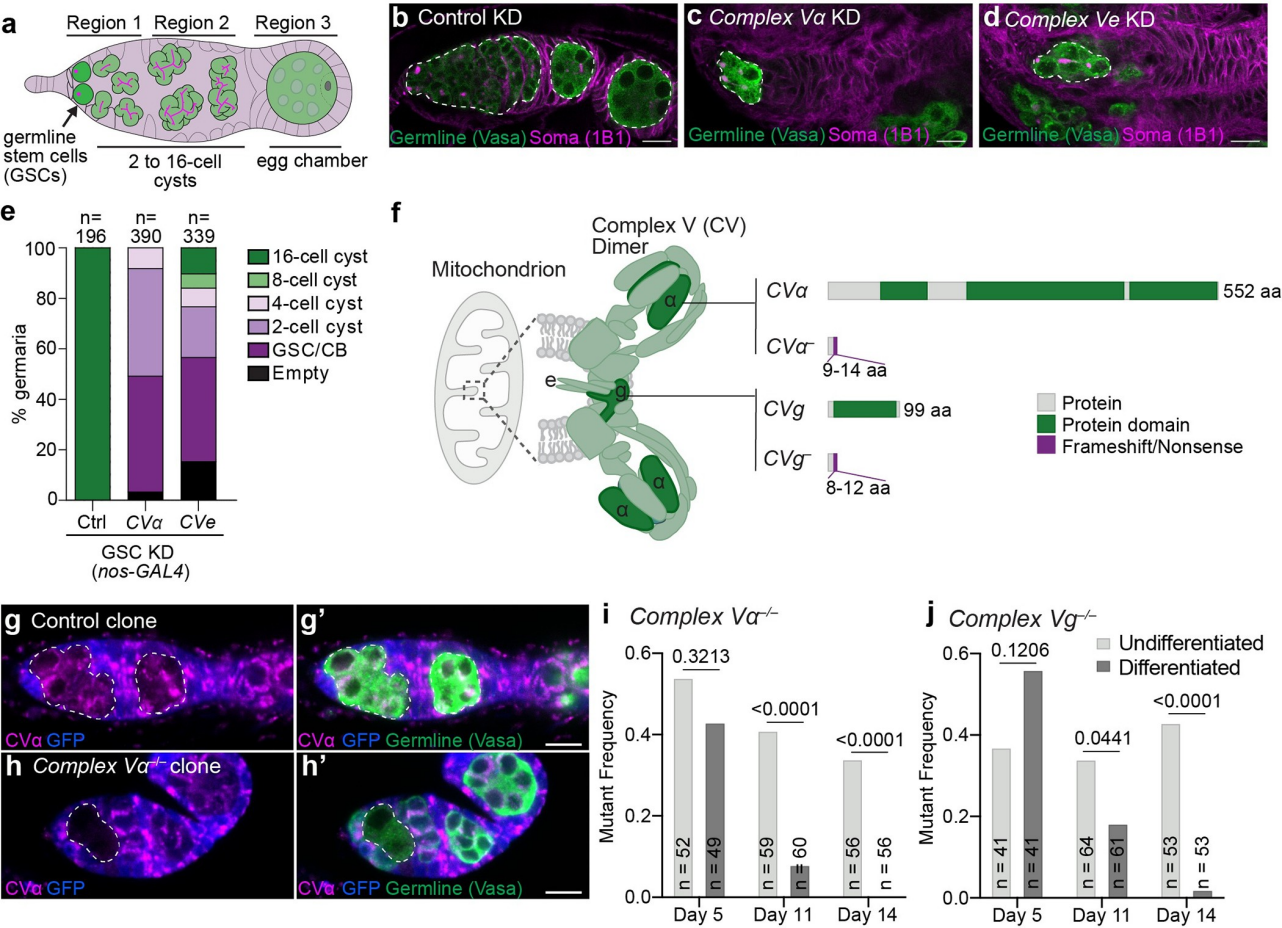
Complex V is essential for germline stem cell differentiation. **(a)** Drosophila germarium. GSCs at the anterior tip of germaria asymmetrically divide and differentiate. The differentiating cell undergoes four rounds of mitosis with incomplete cytokinesis to generate a 16-cell interconnected cysts. The 16-cell cyst buds off as an egg chamber to further mature into an egg. **(b-d)** Representative images of 1-day old Control (*mCherry*) **(b)**, *CVα* **(c)**, and *CVe* **(d)** KD germaria driven by *nos-GAL4*. White-dashed lines mark the germline. **(e)** Quantification of the latest differentiation stage in germaria of indicated genotypes. RNAi were driven by *nos-GAL4*. Number of germaria scored is indicated above each bar. **(f)** Complex V dimer at the tip of mitochondrial cristae. CVα forms one of the main catalytic components of the complex. Subunits g and e are required for dimerization. Putative null alleles in *α* and *g* were generated. **(g, h)** Representative images of Control **(g)** and *CVα* **(h)** mosaic germaria 14-days after clone induction. Mutant GFP-negative clones are marked with the white-dashed lines. **(i, j)** Frequency of *CVα* **(i)** and *CVg* **(j)** mutant clones 5-, 11- or 14-days after clone induction. Undifferentiated: germline cells in region 1 including GSC, cystoblasts, 2-, 4- and 8-cell cysts. Differentiated: germline cells in region 2 containing 16-cell cysts. Number of germaria observed are given inside the bars. P-values were calculated using Fisher’s exact test. For all images scale bars represent 10 μm. For exact genotypes see S2 Table.

We previously identified an unexpected role for Complex V in the early stages of GSC differentiation [12]. In Drosophila, GSC differentiation begins when a GSC differentiates into a cystoblast and undergoes four rounds of division to form a 2, 4, 8 and eventually a terminally differentiated 16-cell interconnected cyst that will develop into an egg (Fig 1A) [17,18]. We found that germline-specific knockdown of Complex V subunits impaired differentiation prior to the 16-cell cyst stage. However, precisely what role Complex V plays in early germ cell differentiation remained unclear.

Complex V has two core functions: (1) to synthesize ATP via oxidative phosphorylation; and (2) to promote cristae formation [13–15]. To determine which of these functions is critical for GSC differentiation, we analyzed germline knockdowns and mutants in *Complex V subunits e* (*CVe*) and *g* (*CVg*), which are required for its cristae activity [14,19], and *Complex V subunit α* (*CVα*), which is required for both its cristae and ATP-synthesizing functions [13]. Knockdown of *CVα* or *CVe* impaired GSC differentiation into 8-cell cysts, with the later stages (8- and 16-cell cysts and beyond) being largely absent when visualized using the germline marker Vasa and the somatic marker 1B1 (Fig 1B–1E). This supports the hypothesis that Complex V’s cristae-forming role is essential for GSC differentiation. Furthermore, we found that knockdown of *CVα* strongly perturbed mitochondrial cristae formation, while knockdown of *CIII* or *CIV* subunits, which are essential for oxidative phosphorylation, did not (S1A–S1D Fig). Together, this indicates that Complex V’s cristae-forming role is essential for GSC differentiation.

To further validate our RNAi observations, we generated presumptive null mutations in *CVα* and *CVg* using CRISPR/Cas9 (Fig 1F). We analyzed Complex V’s role in GSC differentiation in mosaic animals containing control cells (GFP-positive) and cells with homozygous *Complex V* mutations (GFP-negative) (Fig 1G–1J). We analyzed ovaries 5, 11 and 14 days after clone induction, reasoning that by 14 days little, if any, Complex V protein would be present, which we confirmed using an antibody against CVα (Fig 1H). By day 14, we observed a near complete absence of terminally differentiated mutant *CVα* and *CVg* cells, indicating that Complex V is required for GSC differentiation (Fig 1I and 1J). Thus, mosaic analysis further supports a role for Complex V in remodelling mitochondria to promote GSC differentiation.

In addition to Complex V, other factors are known to be critical for cristae biogenesis and maintenance, including Opa1 and Prohibitin (Phb) 1 and 2. Opa1 has been shown to be a critical regulator of cristae across species [20], independent of its role in fusion [21]. Phb1 and 2, which are obligate hetero-oligomers, have been implicated in promoting cristae through both Opa1-dependent [22] and -independent [23,24] mechanisms. We therefore tested if Opa1 and Phb1/2, like Complex V, are necessary for GSC differentiation (S2A–S2D Fig). We found that knockdown of *Opa1*, *Phb1* or *Phb2* resulted in a similar block in germ cell differentiation as *Complex V* knockdown (S2 Fig) and altered cristae (S1E Fig). This further supports a role for mitochondrial remodelling in germ cell differentiation.

To determine if Complex V’s function in early germline development is confined to GSC differentiation, we also assessed its role in GSC proliferation. To focus specifically on GSCs, we inhibited differentiation by mutating the differentiation factor *bam*, causing ovaries to accumulate GSCs and their immediate progeny (cystoblasts) as GSCs continue to divide and proliferate without differentiation [25]. We found that Complex V was not strictly required for GSC proliferation as double *Complex V* knockdown, *bam* mutant ovaries had significantly more GSCs and cystoblasts than *Complex V* knockdown ovaries alone (S3A–S3E Fig). However, fewer germ cells were present in double *Complex V* knockdown, *bam* mutant ovaries compared with *bam* mutant ovaries suggesting that knockdown of *Complex V* does reduce GSC proliferation rate (S3E Fig). Together, our phenotypic analysis demonstrates that Complex V-mediated mitochondrial remodelling is essential for GSC differentiation. We also find that Complex V plays a non-essential role in GSC proliferation.

### Complex V does not regulate germline stem cell differentiation via the permeability transition pore

In addition to its function in ATP generation and mitochondrial remodelling, Complex V has recently been implicated as a major constituent of the permeability transition pore (PTP) [26–29]. The PTP is a putative, nonspecific pore that forms in the inner mitochondrial membrane causing mitochondrial swelling and often cell death [29–31]. It has also been implicated in a range of other processes including the remodelling of mitochondria during myocyte differentiation [32].

The role of Complex V in the PTP is a matter of debate, with some arguing that Complex V subunit c (CVc) rings form a large ion pore when they are uncoupled from the hydrophilic F_1_ part of the complex (S4A Fig) [26–28,33,34], for example upon *CVα* loss. Alternatively, others have argued that the pore comprises Complex V dimers (S4A Fig), and thus would be inhibited by the loss of *CVe* or *CVg* [35]. Still others have argued that the pore does not contain Complex V subunits at all [36]. Thus, loss of *CVα*, *CVe* or *CVg* could either activate or inhibit the PTP or have no effect on the PTP at all.

To test if the effects of Complex V on GSC differentiation are mediated by PTP opening, we overexpressed the PTP regulator cyclophilin D [30] (S4A Fig). Although Drosophila encode 14 cyclophilins, no mitochondrial cyclophilin has been identified thus far in fruit flies, unlike in yeast and mammals [37]; however, human cyclophilin D can regulate the PTP when it is expressed in Drosophila cells [38]. Consistent with human cyclophilin D promoting the PTP in *Drosophila*, we observed swelling of germline mitochondria by transmission electron microscopy in cyclophilin D expressing ovaries (S4B and S4C Fig). Despite this, no impairment in GSC differentiation was observed when human cyclophilin D was overexpressed (n = 150) (S4D–S4H Fig). Therefore, we conclude that PTP activation does not inhibit GSC differentiation.

To further test if Complex V perturbation impairs differentiation by promoting PTP opening, we assessed the effect of *CVc* knockdown on differentiation, as CVc is required for PTP opening in all Complex V-based models. If knockdown of other *Complex V* subunits inhibits differentiation by inducing PTP opening, knockdown of *CVc* should have the opposite effect and not inhibit differentiation. In contrast to this prediction, we found that *CVc* knockdown inhibited differentiation (S4I Fig) similar to *CVα* or *CVe* knockdowns (Fig 1C and 1D). Therefore, together this suggests that *Complex V* loss does not impair GSC differentiation through activation of the PTP. Furthermore, our data neither support nor refute a role for Complex V in the PTP in Drosophila.

### Germ cells undergo a metabolic rewiring during germline development

The formation of mitochondrial cristae is thought to increase the surface area of the inner mitochondrial membrane, allowing for the accommodation of more oxidative phosphorylation complexes and thus greater mitochondrial ATP generating capacity [39,40]. Inner mitochondrial membrane remodelling during differentiation may thus serve to equip mitochondria with the ability to host more oxidative phosphorylation complexes and thus generate large quantities of ATP to support differentiation. If this is the case, we would expect GSC differentiation to be blocked when oxidative phosphorylation is inhibited. Thus, we assessed whether loss-of-function mutations in core subunits of the oxidative phosphorylation Complexes II, III and IV influence germ cell development (S5A and S5B Fig). Remarkably, mosaic analysis with loss-of-function mutations in *Complex II subunit D*, *Complex III subunit Cyt-c1* or *Complex IV subunit 5A* showed GSCs differentiated to the 16-cell cyst stage (S5C–S5I Fig), even though mitochondrial membrane potential was severely impaired in Complex III and IV homozygous mutant clones (S5E and S5F Fig). These data suggest that core subunits of Complexes II, III and IV are not required for early germ cell development. We conclude GSC differentiation does not strongly require oxidative phosphorylation [12].

**Fig 2:**
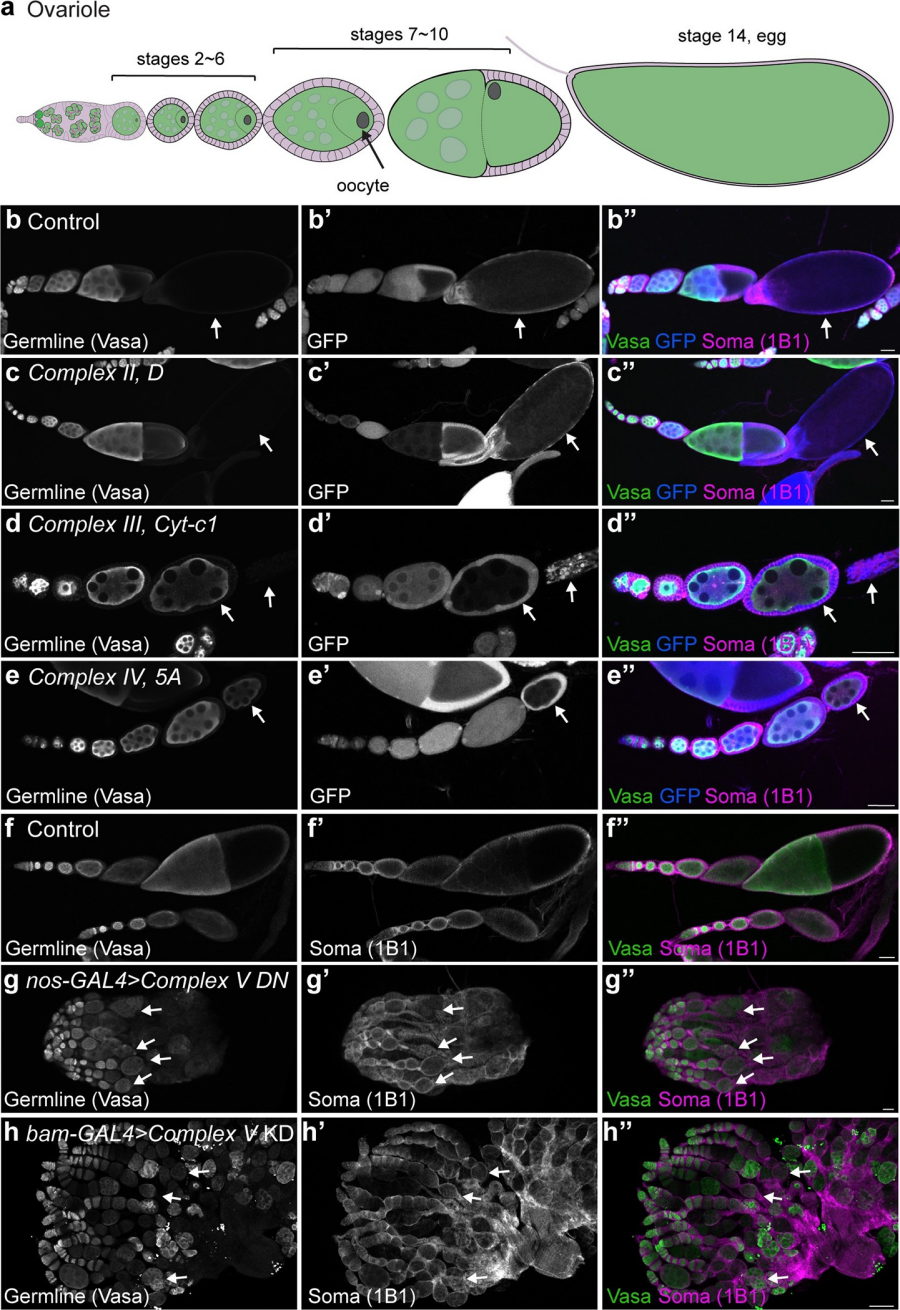
Egg development requires mitochondrial oxidative phosphorylation. **(a)** Schematic of an ovariole. An egg chamber is comprised of one oocyte, fifteen nurse cells and surrounding somatic follicle cells. Oogenesis comprises 14 stages resulting in the formation of a mature egg. **(b-e)** Representative images of Control **(b)**, *Complex II subunit D* **(c)**, *Complex III Cyt-c1* **(d)** and *Complex IV subunit 5A* **(e)** mosaic ovarioles 14-days post-clone induction. Arrows indicate GFP-negative mutant cells. **(f, g)** Representative images of 2–3 day old Control **(f)** and germline expressed (*nos-GAL4*) *Complex V dominant negative (DN)* **(g)** in heterozygous *CVc* deficiency ovaries. **(h)** Representative image of a 2–3 day old *bam-GAL4* driven *CVα* RNAi ovary. Arrows indicate the last stage observed in the representative ovariole. For all images scale bars represent 100 μm. For exact genotypes see S2 Table.

Mitochondrial remodelling could still act to endow mitochondria with the necessary ATP generating capacity to support later germline development. To explore this, we asked if egg chamber development was perturbed in *Complex II*, *III* and *IV* mutant animals. Egg chambers are the equivalent of mammalian ovarian follicles, which grow and develop over the course of several days to give rise mature eggs [17] (Fig 2A). While egg chamber development proceeded normally and mature eggs were produced in control animals (Fig 2B), this was profoundly impaired in *Complex III* or *Complex IV* mutants with no development past stage 9 observed (n>48) (Fig 2D–2E). Consistent with this, specifically inhibiting Complex V’s ATP synthesizing activity, by overexpressing a dominant negative CVc [41] in a heterozygous deficiency background, impaired egg chamber development, but not earlier germline development (Fig 2F and 2G). A similar phenotype was also observed when *CVα* was knocked down after terminal differentiation using the *bam-Gal4* driver (Fig 2H). Surprisingly, we did not observe defects in egg chamber development in *Complex II mutants* suggesting that Complex I may compensate for loss of Complex II as they work in parallel (Fig 2C). Therefore, we conclude that in contrast to earlier stages of development, mitochondrial oxidative phosphorylation is required for egg chamber growth and survival.

Together, our data demonstrate that germ cells do not rely strongly, if at all, on mitochondria to make ATP during their early stages of differentiation but undergo a metabolic rewiring, becoming dependent on oxidative phosphorylation for egg chamber development. Therefore, mitochondrial remodelling is not required to generate ATP for GSC differentiation but may serve to equip mitochondria with the capacity to generate enough ATP to support later egg production.

### Impairment of mitochondrial remodelling activates the Integrated Stress Response

**Fig 3:**
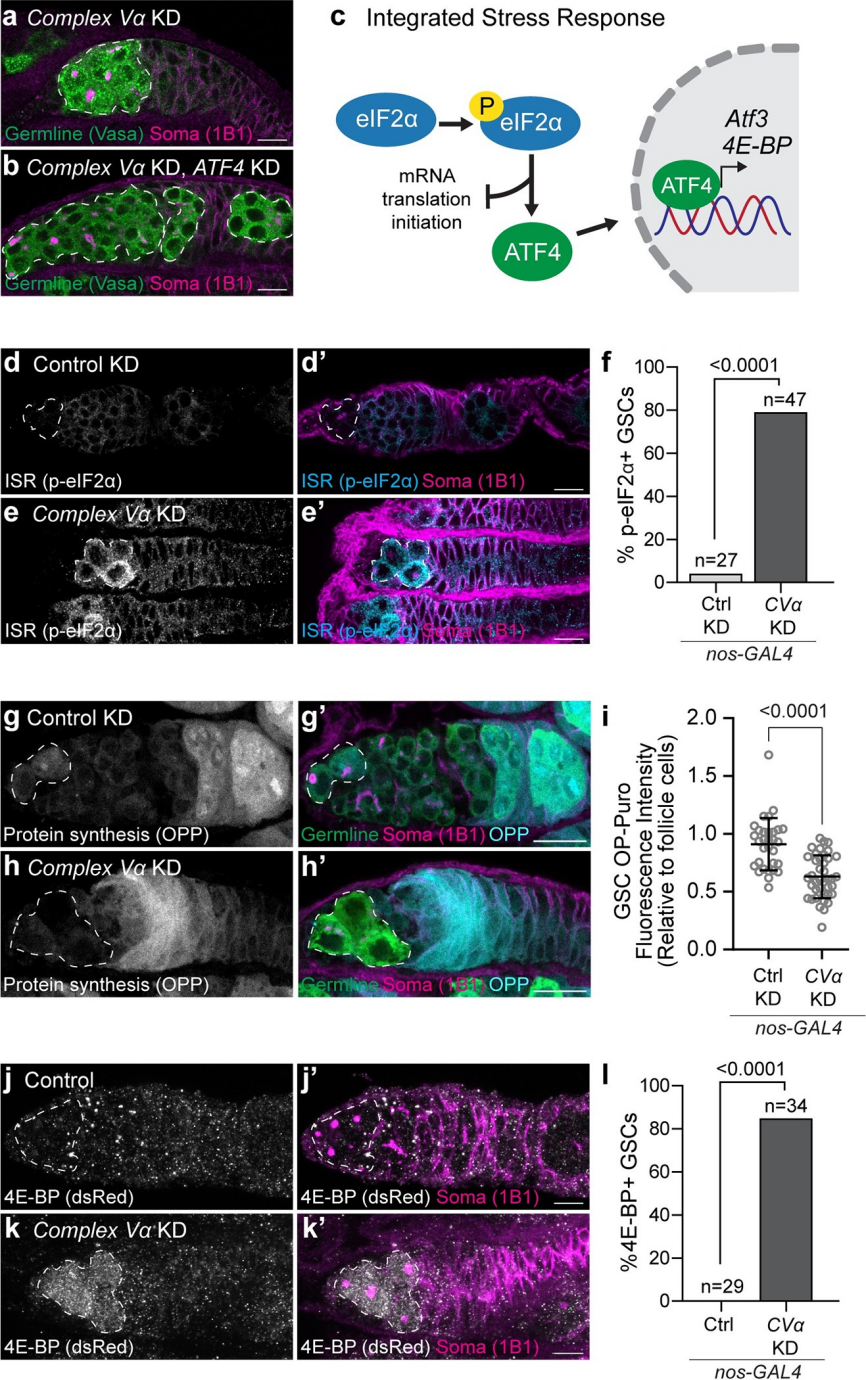
Complex V knockdown activates the Integrated Stress Response. **(a, b)** Representative images of 2–3 day old *CVα* KD **(a)**, and *CVα* and *ATF4* KD **(b)** germaria. **(c)** Schematic of the Integrated Stress Response (ISR). Phosphorylation of eIF2α activates the ISR by inhibiting global cap-dependent translation and selectively upregulating the translation of the transcription factor, ATF4. ATF4 upregulates the transcription of stress response genes including *4E-BP* and *Atf3*. **(d, e)** Representative images of 2–3 day old Control (*mCherry*) **(d)** and *CVα* **(e)** KD germaria. **(f)** Frequency of GSCs with high phosphorylated eIF2α relative to surrounding somatic cells from **(d, e)**. **(g, h)** Representative images of 1-day old Control (*mCherry*) **(g)** and *CVα* **(h)** KD germaria incubated with O-propargyl-puromycin (OPP) to visualize protein synthesis. **(i)** Intensity of fluorescently derivatized OPP in GSCs relative to follicle cells of Control (*mCherry*) KD (n = 31) and *CVα* KD (n = 40). Data are the mean ± s.d. and an unpaired t-test was used for statistical analysis. **(j, k)** Representative images of less than one week old Control **(j)** and *CVα* **(k)** KD germaria expressing ATF4 reporter, *4E-BP*^*intron*^*-*dsRed. **(l)** Frequency of dsRed-positive GSCs in **(g, h).** For **(f, l)** number of GSC analyzed and P-value (Fisher’s exact test) are given above bars. For **(a, b)** white-dashed lines demark the germline, and for **(d-e, g-h, j-k)** white-dashed line demark GSCs. All RNAi were driven by *nos-GAL4*. For exact genotypes see S2 Table.

If increased ATP production is necessary to support later development, then why is GSC differentiation compromised when mitochondrial remodelling is inhibited? To explore this, we conducted a targeted suppressor screen to determine if aberrant regulation of stress pathways might be inhibiting GSC differentiation. We screened known mitochondrial-to-nuclear retrograde signalling factors including AMPK, the JNK-FOXO pathway, the Unfolded Protein Response (UPR), and the Integrated Stress Response (ISR) [1]. Knockdown or loss of *AMPKα*, *foxo* or the UPR component *Ire1* did not restore germ cell differentiation in *CVα* knockdowns (S6A–S6E Fig), nor did overexpression of *foxo*. Furthermore, we did not observe activation of these pathways in *CVα* knockdowns (S6F–S6L Fig). Interestingly, however, knockdown of the transcription factor *ATF4*, a central component of the ISR, rescued GSC differentiation in *Comple*x V knockdowns (Figs 3A, 3B and S6A). Nearly all *CVα*, *ATF4* double knockdown ovaries possessed egg chambers (S6A Fig), which are never observed in *CVα* only knockdowns (Fig 1E). Thus, we conclude that impairment of mitochondrial remodelling activates the ISR.

The hallmark of ISR activation is the phosphorylation of eIF2α, which inhibits global mRNA translation initiation but selectively enhances translation of ATF4 and downstream targets [42,43] (Fig 3C). If *Complex V* knockdown activates the ISR, we would expect to observe: (1) an increase in eIF2α phosphorylation; (2) a decrease in global mRNA translation; and (3) an increase in ATF4 activity. Consistent with this, we found that *CVα* knockdown germ cells had elevated levels of phosphorylated eIF2α compared to the surrounding somatic cells, and in contrast to wildtype germ cells which had little to no detectable phosphorylated eIF2α (Fig 3D–3F). eIF2α phosphorylation was also increased when mitochondrial remodelling was perturbed by knockdown of *Opa1* or *Phb1* (S7A–S7F Fig). We found that in *CVα* knockdowns there was a reduction in protein synthesis as measured using O-propargyl-puromycin [44,45] (Fig 3G–3I). Lastly, we found that *CVα* knockdown increased ATF4 activity as measured using a *4E-BP* transcriptional reporter, which is known to be upregulated by ATF4 [46] (Fig 3J–3L) and *Atf3* mRNA expression, a known ATF4 target gene [47,48] (S6J Fig). Taken together, these data indicate that defects in mitochondrial remodelling prevent GSC differentiation through activation of the ISR.

### Impairment of mitochondrial remodelling activates PERK

**Fig 4:**
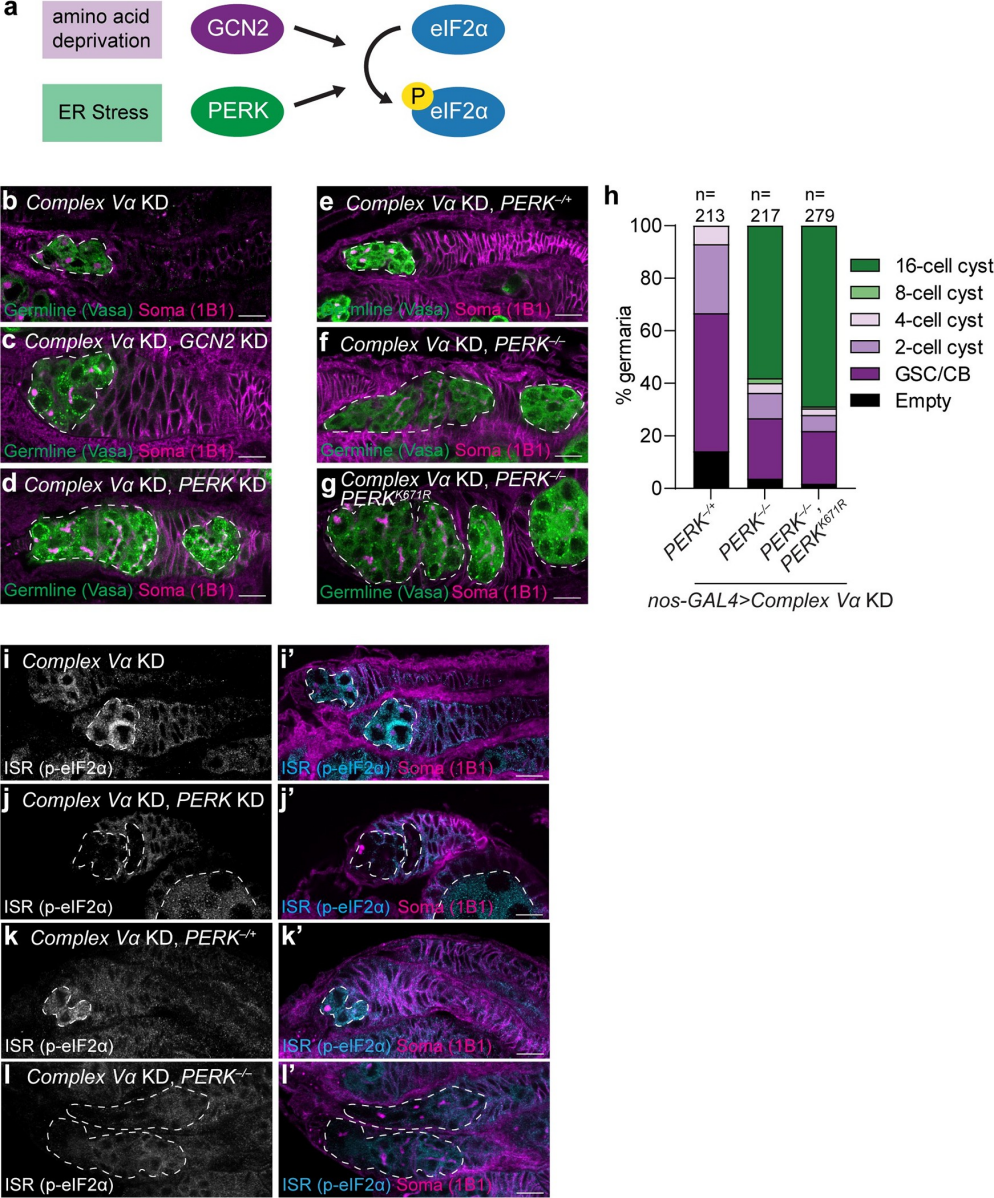
Complex V knockdown activates the Integrated Stress Response through PERK. **(a)** Schematic of *Drosophila* eIF2α kinases. Amino acid deprivation or endoplasmic reticulum (ER) stress activate either GCN2 or PERK, respectively. Both kinases dimerize and phosphorylate eIF2α. **(b-d)** Representative images of 2–3 day old *CVα* KD **(b)**, *CVα*, *GCN2* KD **(c)**, and *CVα*, *PERK* KD **(d)** germaria. **(e-g)** Representative images of 1 day old *CVα* KD, *PERK*^*–/+*^ **(e)**, *CVα* KD, *PERK*^*–/–*^**(f)**, and *CVα* KD, *PERK*^*–/–*^, *UAS-PERK*^*K671R*^ **(g)** germaria. **(h)** Phenotypic characterization and quantification of germline differentiation stage in germaria of **(e-g)**. Number of germaria scored above each bar and were studied from 10 or more ovaries, obtained in at least three independent experiments and over two or more crosses. **(i-l)** Representative images of 2–3 day old *CVα* KD **(i)**, *CVα*, *PERK* KD **(j)**, *CVα* KD, *PERK*^*–/+*^ **(k)** and *CVα* KD, *PERK*^*–/–*^**(l)** germaria. All RNAi were driven by *nos-GAL4*. Images are representative of over 100 ovarioles and three independent experiments. Scale bars represent 10 μm. White-dashed line indicates the germline cells. For exact genotypes see S2 Table.

In Drosophila, the ISR responds to a wide variety of stresses through two conserved kinases, GCN2 and PERK [46,49–52] (Fig 4A). GCN2 senses amino acid deprivation while PERK, an ER membrane protein, is activated by misfolded proteins in the ER and ER lipid bilayer stress [42,53,54]. When activated, both kinases dimerize and phosphorylate eIF2α leading to global protein synthesis attenuation, while also enhancing translation of stress response genes [42]. To determine which kinase mediates ISR activation upon *Complex V* knockdown, we asked whether knockdown of *GCN2* or *PERK* would rescue the differentiation defect caused by *Complex V* knockdown. Interestingly, we found that knockdown of *PERK* partially rescued differentiation of GSCs in *Complex V* knockdowns to a similar degree as *ATF4* knockdown, whereas knockdown of *GCN2* had no effect, despite highly efficacious silencing (Figs 4B–4D and S8A–S8E). Importantly, *PERK* knockdown did not restore fertility in *Complex V* knockdowns as egg chambers failed to grow and develop (S8E Fig) consistent with a later function for Complex V-generated ATP in egg development (Fig 2).

To confirm our *PERK* knockdown results, we made two unique *PERK* loss-of-function mutant alleles, *PERK*^*1*^ and *PERK*^*2*^, that are predicted to generate truncated PERK lacking all the characterized PERK domains including its lumenal, transmembrane and kinase domains (S8F Fig) [49]. *PERK* transheterozygous mutant (*PERK*^*1/2*^) flies did not have an apparent female fertility defect and laid viable eggs (S8G and S8H Fig). Consistent with our previous analysis, knockdown of *CVα* in a *PERK* transheterozygous mutant background partially rescued GSC differentiation resulting in ovaries that develop into 16-cell cysts (Fig 4E–4F and 4H). Both knockdown and deletion of *PERK* prevented eIF2α phosphorylation caused by *Complex V* knockdown (Fig 4I–4L). Furthermore, the differentiation defect was dependent on PERK’s kinase activity as reintroduction of a kinase dead PERK (PERK^K671R^) [49,55] in a *PERK*^*1/2*^ mutant background did not prevent differentiation (Fig 4G and 4H). Together, this indicates that *Complex V* knockdown activates PERK, which phosphorylates eIF2α leading to increased translation of ATF4 and inhibition of GSC differentiation.

### Impairment of mitochondrial remodelling induces endoplasmic reticulum lipid bilayer stress

Given its localization, how defects in mitochondrial remodelling activate PERK remains unclear. PERK resides in the ER membrane where it senses both lipid bilayer stress and misfolded proteins [53,56–58]. As phospholipids from the ER are necessary for cristae formation [59,60], we questioned if impairment of cristae biogenesis by *Complex V* knockdown might perturb lipid transfer between the ER and mitochondria resulting in ER lipid disequilibrium, PERK activation and GSC differentiation defects.

PERK senses lipid bilayer stress through its transmembrane domain and lumenal misfolded proteins via its lumenal domain [53]. We generated a PERK mutant lacking the lumenal domain (*PERK*^*ΔLD*^) (S9A and S9B Fig), which is unable to sense protein misfolding but is still sensitive to lipid bilayer stress [53]. The removal of the lumenal domain did not alter PERK^ΔLD^ protein expression or ER localization (S9C–S9G Fig). It was not possible to generate PERK mutants that are unable to sense lipid bilayer stress as PERK’s transmembrane domain can tolerate a range of amino acid substitutions without abolishing its lipid sensing function [53].

**Fig 5:**
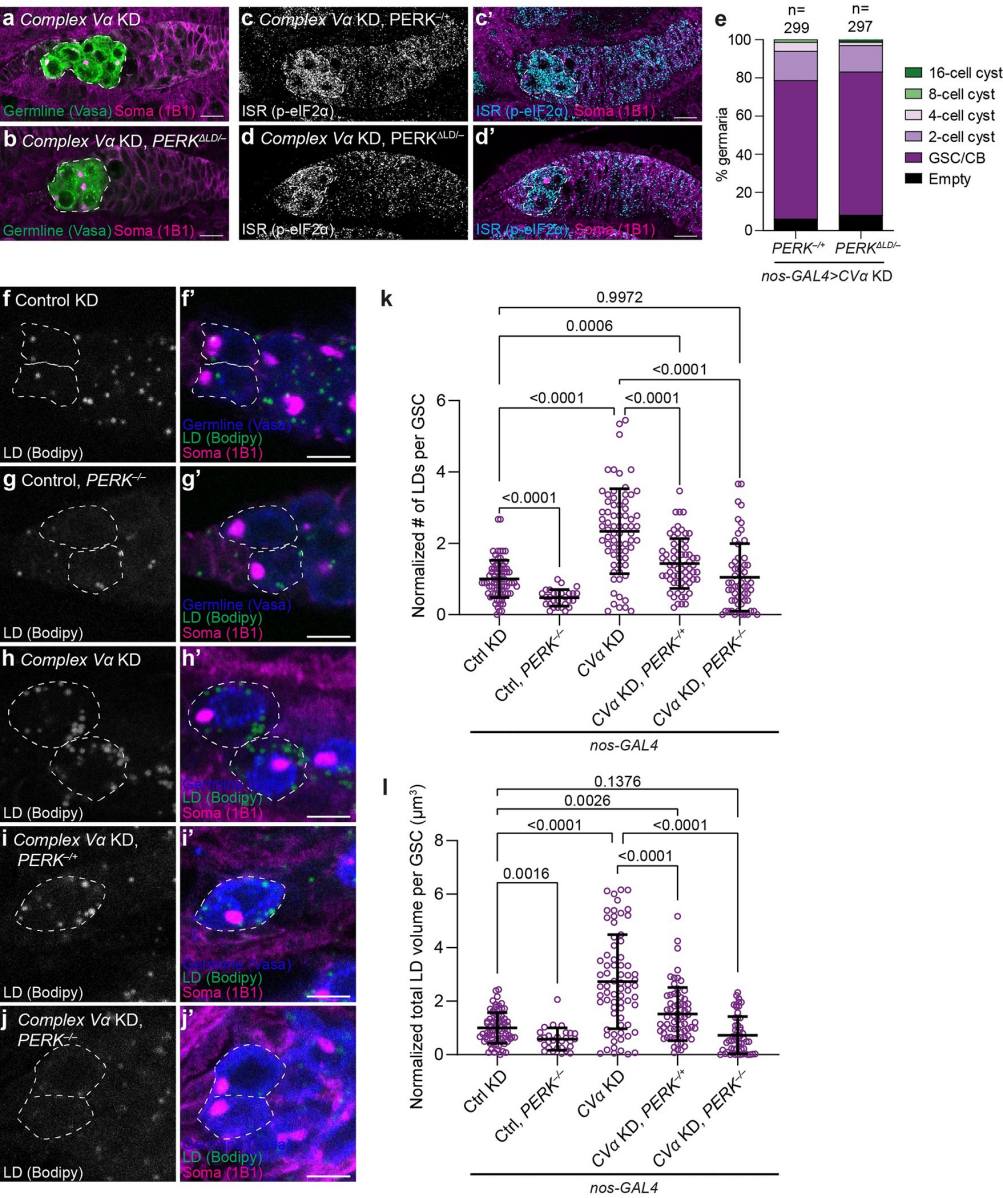
Complex V knockdown induces endoplasmic reticulum lipid bilayer stress. **(a-d)** Representative images of 1-day old *CVα* KD, *PERK*^*–/+*^ **(a, c)** and *CVα* KD, *PERK*^*ΔLD/–*^**(b, d)** germaria. GSCs are outlined in white-dashed lines. **(e)** Quantification of germline differentiation stage in germaria of indicated genotypes. Number of germaria scored above each bar. **(f-j)** Representative images of 2–3 day old Control (*mCherry)* KD **(f)**, *PERK*^*–/–*^**(g)**, *CVα* KD **(h)**, *CVα* KD, *PERK*^*–/+*^ **(i)** and *CVα* KD, *PERK*^*–/–*^**(j)** GSCs (white-dashed line). BODIPY 493/503 marks lipid droplets. **(k)** Quantification of number of lipid droplets per GSC normalized to mean of Control for the indicated genotypes (*n* = 75 for Ctrl (*mCherry*) KD; *n* = 28 for Ctrl, *PERK*^*–/–*^; *n* = 71 for *CVα* KD; *n* = 68 for *CVα* KD, *PERK*^*–/+*^; and *n* = 59 for *CVα* KD, *PERK*^*–/–*^). **(l)** Quantification of total lipid droplet volume per GSC normalized to the mean of the Control for the indicated genotypes (*n* = 73 for Ctrl (*mCherry*) KD; *n* = 28 for Ctrl, *PERK*^*–/–*^; *n* = 72 for *CVα* KD; *n* = 68 for *CVα* KD, *PERK*^*–/+*^; and *n* = 55 for *CVα* KD, *PERK*^*–/–*^). All RNAis were driven by *nos-GAL4*. Scale bars represent 10 μm **(a-d)** and 5 μm **(f-j)**. For all plots, germaria were studied from 10 or more ovaries, obtained in at least three independent experiments and over two or more crosses. For **(k, l)**, data are mean ± s.d. and one-way ANOVA followed by Games-Howell multiple comparison’s test was used for statistical analysis. For exact genotypes see S2 Table.

*PERK*^*ΔLD*^ mutant analysis supported the hypothesis that impairing mitochondrial remodelling activates PERK by inducing ER lipid bilayer stress. In contrast to *PERK* deletion mutants, we found that *PERK*^*ΔLD*^ mutants did not rescue the differentiation defect indicating that *Complex V* knockdowns are still capable of activating a form of PERK that is unable to sense protein misfolding (Fig 5A–5E). Reactive oxygen species have also been implicated in PERK activation [61,62]; however, we observed no increase in hydrogen peroxide in *CVg* null mutant clones (S10A and S10B Fig), and no increase in the oxidative stress reporter, *GstD1-GFP* [63], in *CVα* or *CVc* knockdowns (S10C–S10E Fig). Thus, we propose that impairing mitochondrial remodelling activates PERK by inducing ER lipid bilayer stress which is sensed through PERK’s lipid sensing transmembrane domain rather than its misfolded protein-sensing lumenal domain.

Lipid droplets have been suggested to act as a buffer against lipid toxicity and correct lipid composition [64]. Therefore, if impairment of mitochondrial remodelling induces an ER lipid bilayer stress, we would expect to observe an increase in lipid droplets in *Complex V* knockdowns. Indeed, we detected a significant increase in the number and total volume of lipid droplets visualized by BODIPY 493/503 in *CVα* knockdowns compared with controls (Fig 5F–5I). Since the ISR has been implicated in promoting lipogenesis [65–68], we wondered whether the increase in lipid droplets was driven by PERK itself. Indeed, the loss of *PERK* prevented lipid droplet accumulation in a dose-dependent manner (Fig 5F–5I). Interestingly, we also found that knockdown of *ATF4* similarly prevents lipid droplet accumulation suggesting that PERK promotes lipids droplets via ATF4 (S11A–S11E Fig). Together this suggests that a failure to remodel mitochondria during GSC differentiation causes ER lipid bilayer stress, which activates PERK leading to the inhibition of differentiation.

### Impairment of mitochondrial remodelling induces precocious meiosis and compromises the survival of differentiating germ cells

**Fig 6:**
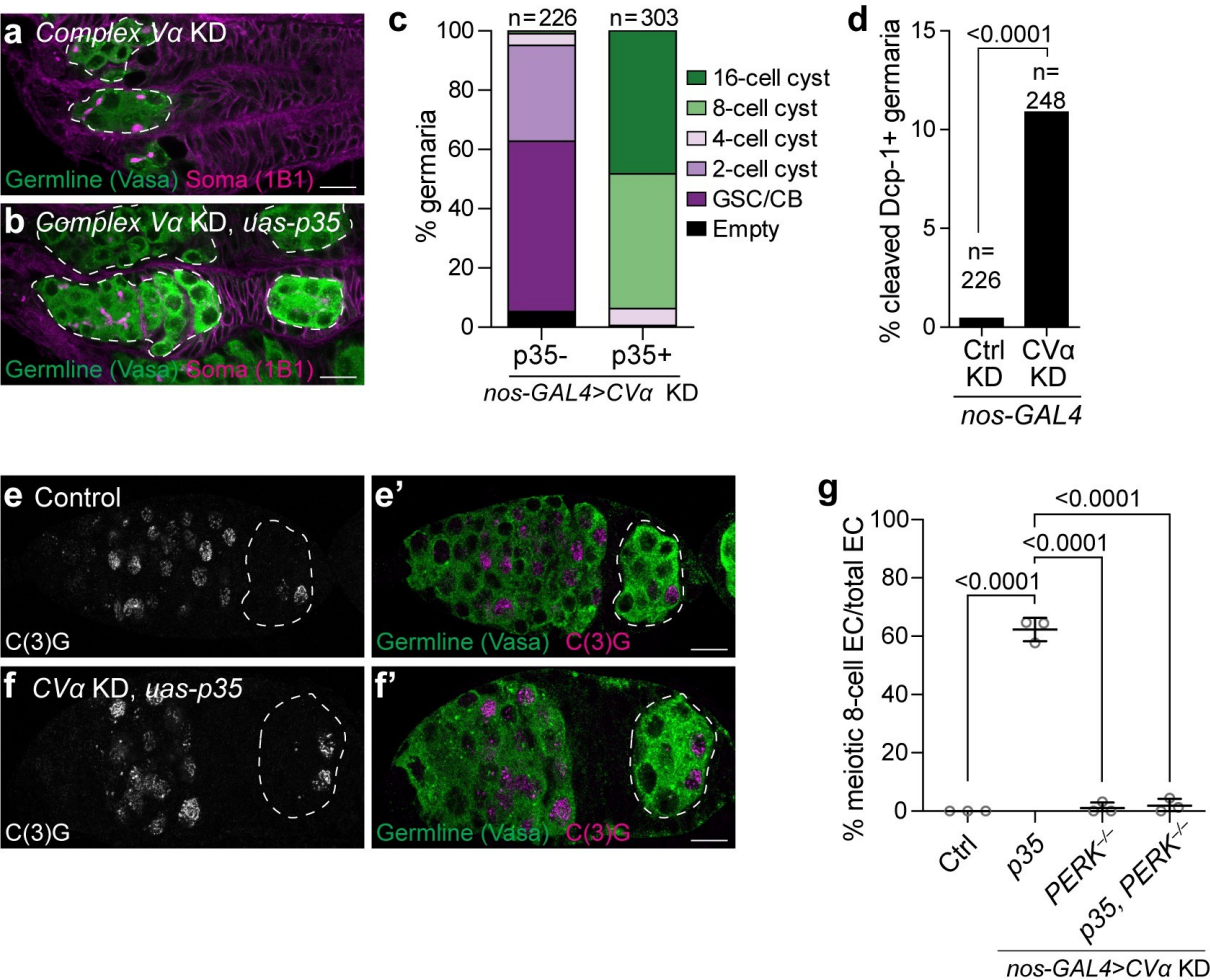
Loss of Complex V induces precocious meiosis and cyst death. **(a, b)** Representative images of 1 day old *CVα* KD **(a)** and *CVα* KD, *P35* overexpression **(b)** germaria. White-dashed lines demarks the germline. **(c)** Phenotypic characterization and quantification of germline differentiation stage in germaria of **(a, b)**. Number of germaria scored above each bar and were studied from 10 or more ovaries, obtained in at least three independent experiments and over two or more crosses. **(d)** Frequency of cell death positive germaria of 2–3-day old ovaries Control (*mCherry*) KD and *CVα* KD immunostained with anti-cleaved Dcp1, which marks cells undergoing apoptosis. Number of ovarioles studied and P-value (Fisher’s exact test) are given above bars. **(e, f)** Representative images of 1 day old Control **(e)** and *CVα* KD, *P35* overexpression **(f)** germaria stained with the synaptonemal complex component C(3)G to mark cells in meiosis. See S12E and S12F Fig for confocal slices highlighting number of nuclei. White-dashed lines demark the Region 3 egg chamber. **(g)** Frequency of meiotic 8-cell cyst egg chambers (EC) for the indicated genotypes (*n* = 79 for Control (*no GAL4*); *n* = 122 for *CVα* KD, *UAS-P35*; *n* = 120 for *CVα* KD, *PERK*^*–/–*^; and *n* = 229 for *CVα* KD, *UAS-P35*, *PERK*^*–/–*^). Each data point represents a replicate with 20–90 egg chambers analyzed and plotted as mean ± s.d. An ANOVA followed by Tukey post-hoc test was performed. All RNAi and P35 were driven by *nos-GAL4*. For all images scale bars represent 10 μm. For exact genotypes see S2 Table.

Impairing mitochondrial remodelling compromises GSC differentiation. We next wondered if the germline cysts were dying and if restoring cyst survival would restore differentiation. To determine if and how cysts die, we first asked if expressing the effector caspase-inhibiting baculovirus protein P35 would prevent cyst death caused by *Complex V* knockdown [69]. Indeed, we found that P35 expression prevented cyst death in *CVα* knockdowns, resulting in egg chambers containing either 8- or 16-cell cysts (Figs 6A–6C, S12A and S12B). In line with the P35 results, we observed an increase in the cell death marker cleaved Dcp-1 in *Complex V* knockdowns compared with controls (Fig 6D). Knockdown of the initiator caspase Dronc (homologous to mammalian caspase-9), the pro-apoptotic protein Hid and apoptosome adaptor protein Dark [70], strongly inhibited cell death, suggesting that *Complex V* knockdown germline cysts undergo apoptosis (S12C Fig). Therefore, inhibition of mitochondrial remodelling causes ER lipid bilayer stress, which leads to the sustained activation of PERK, the ISR and apoptosis of differentiating germ cells.

While overexpression of P35 prevented cell death in *Complex V* knockdowns, it did not largely restore differentiation, as nearly half of the cysts failed to differentiate to the 16-cell cyst stage (Fig 6C). This indicates that Complex V has an important role in differentiation beyond maintaining cyst survival. One way that differentiation from the 8- to 16-cell cyst stage can be blocked is if 8-cell cysts undergo a premature mitotic-to-meiotic transition, which, interestingly, has previously been associated with cell death [71]. Thus, we asked if *Complex V* knockdown induces a premature meiotic entry. We assessed meiotic entry in control and *Complex V* knockdowns by imaging the synaptonemal complex protein C(3)G, which appears threadlike when pro-oocytes are arrested in pachytene [71–73]. To increase the proportion of cysts, we simultaneously inhibited cell death by overexpressing P35. We found that all 8-cell egg chambers in *Complex V* knockdown ovaries had prematurely entered meiosis. We never observed a similar meiotic pachytene configuration in wildtype 8-cell cysts (Figs 6E–6G, S12D and S12E). Furthermore, this premature meiotic phenotype was not observed in double *Complex V* knockdown, *PERK*^*1/2*^ mutants (Fig 6G), indicating that it is dependent on the ISR. Thus, our data suggest that Complex V plays a critical role in the timing of meiotic entry during GSC differentiation.

## Discussion

Among the most striking cytological features of stem cells is that their mitochondria often appear immature with few cristae, which increase in number as stem cells differentiate. Here, we show, that Complex V plays a key role in this process. Our data suggest that Complex V drives the remodelling of the inner mitochondrial membrane during the early stages of GSC differentiation to increase the ATP generating capacity of mitochondria to support the later growth and development of eggs. Intriguingly, even though early germ cells do not require cristae and mitochondria for much, if any, ATP production, mitochondrial remodelling is still nevertheless essential for differentiation at these stages. Probing this conundrum, we find that a failure to remodel mitochondria results in lipid bilayer stress in the ER that is sensed by PERK, which induces the ISR, premature meiosis and cell death in differentiating germ cells. Thus, perturbing one aspect of a developmentally regulated mitochondrial remodelling program, the formation of cristae in the inner mitochondria membrane, can result in untenable imbalances in other aspects of the program (S13 Fig).

### Mitochondrial remodelling during differentiation

Mitochondria undergo alterations as cells differentiate to meet the changing requirements that emerge upon differentiation. Indeed, recent studies have documented significant remodelling of the mitochondrial proteome and inner mitochondrial membrane during differentiation [74–78]. The factors that drive these changes in mitochondrial content and structure during cellular differentiation, however, remain poorly characterized particularly in *in vivo* contexts. Here, we demonstrate a role for Complex V in remodelling mitochondria during germ cell differentiation. In addition to Complex V, which creates positive curvature at the tips of cristae, we found that Opa1 and Prohibitin1/2, which have been implicated in cristae maintenance and biogenesis [79], also to be similarly involved in germ cell differentiation. A third complex, MICOS, is critical for cristae junction formation [80]. Determining to what degree the MICOS complex contributes to mitochondrial remodelling during germ cell differentiation remains an important avenue of future inquiry.

Alternative mechanisms have been proposed to promote mitochondrial remodelling during differentiation in different cell types and species. Closure of the PTP has been shown to facilitate cardiac mitochondrial remodelling and myocyte differentiation [32]; however, we found this not to be required for differentiation of Drosophila germ cells. PERK has also recently been implicated in promoting cristae formation by increasing import of the MICOS complex subunit, MIC19, into mitochondria during cold-induced brown adipose tissue differentiation [75,76]. Interestingly, while we found PERK to be dispensable for Drosophila germ cell differentiation, PERK was activated by *Complex V* loss suggesting that crosstalk between the mitochondria and ER is critical during cellular differentiation.

### Mitochondrial energy metabolism in the Drosophila germline

Changes in mitochondrial structure during differentiation are thought to reflect changes in energy metabolism with differentiated cells often being more dependent on oxidative phosphorylation than undifferentiated ones [2]. Indeed, our data is consistent with this idea, as we found that Drosophila female germ cells undergo a metabolic transition becoming strongly dependent on oxidative phosphorylation for oocyte growth and development. Consistent with a transition in energy metabolism, oxidative phosphorylation complexes are transcriptionally upregulated after terminal cyst differentiation in the female Drosophila germline [81]. Thus, the increased surface area accompanying mitochondrial remodelling may provide critical inner mitochondrial membrane space in which to place these additional oxidative phosphorylation complexes. During late egg chamber growth and development large quantities of RNA and proteins are synthesized and deposited into the developing oocyte [17,82], perhaps explaining the increased requirement for efficient ATP production via oxidative phosphorylation at these stages.

Earlier stages of oogenesis, however, were remarkably oxidative phosphorylation independent. What energy sources fuel germ cells at these earlier stages remains unclear. Previous expression profiling of purified GSC indicates that arginine kinase is highly expressed in GSCs and their immediate progeny suggesting that they may employ an arginine-phosphate and arginine kinase phosphagen system to store and utilize energy imported from neighboring cells [83]. As the elimination of deleterious mitochondria DNA occurs at these early stages [41,84,85], uncoupling development from mitochondrial energy production may facilitate the process of selection without affecting germline development.

### Mitochondria and germ cell death

Even though germ cells are not dependent on oxidative phosphorylation for early differentiation, Complex V was still required at these stages. We found that loss of Complex V activates the PERK-eIF2α-ATF4 pathway triggering cell death. Why differentiating germ cells die and not GSCs remains to be determined. One possibility is that there is an increase in the production of precursor lipids in the ER to support cristae biogenesis [59] during differentiation, and that inhibiting cristae formation, but not lipid production, may thus result in increased ER lipid bilayer stress at these stages. Consistent with this, we observed a significant increase in lipid droplets in *Complex V* knockdowns. Alternatively, *Complex V* knockdown germ cells might die during differentiation because they are more vulnerable to cell death than GSCs. Consistent with this, differentiating germline cysts have been shown to more sensitive to spontaneous, starvation and irradiation-induced cell death than GSCs [86–88]. Lastly, we found that *Complex V* knockdown induces a premature mitotic-to-meiotic transition, which may in turn induce cell death for reasons that remain to be determined. Consistent with this, Tor kinase mutants, which undergo a similar premature meiotic entry, also undergo cyst cell death [71].

The intrinsic factors regulating cell death at these stages in the female germline remain very poorly understood. Our data implicates PERK in promoting germ cell death via the classical apoptotic *hid*-*dronc/dark* pathway. Drosophila Hid (head-involution defect) localizes to the mitochondria and antagonizes Inhibitor of Apoptosis Proteins (IAPs), which makes it well placed to induce apoptosis caused by *Complex V* knockdown [89]. Similar to what has been observed in the Drosophila testis, we implicated Dronc in the female germ cell death; however, unlike the testis where Dronc non-canonically promotes necrotic germ cell death [90], we find that in female germline cysts Dronc likely acts canonically to induce apoptosis. Precisely how PERK actives apoptosis in the female germline remains to be determined; however, it may involve the direct transcriptional downregulation of the Death-associated inhibitor of apoptosis 1 (DIAP1) [91] or regulate other targets of the apoptotic pathway.

### Mitochondria and meiotic entry

Differentiating germ cells normally undergo four rounds of mitosis to form a 16-cell cyst prior to entering meiosis and terminally differentiating. Interestingly, we observed that *Complex V* knockdown germ cells enter meiosis precociously suggesting that Complex V may be necessary for regulating the timing of meiotic entry. We further found that knockout of PERK prevents the precocious meiosis phenotype in *Complex V* knockdowns suggesting that activating the ISR triggers a precocious mitotic-to-meiotic transition. The signals controlling the mitotic-to-meiotic transition are not well understood in Drosophila; however, protein synthesis is sharply reduced at the onset of meiotic entry [44]. One intriguing possibility is that activation of ISR and the ensuing global reduction in protein synthesis is sufficient to induce meiotic entry.

### Mitochondria, PERK and the ISR

The ISR is a key regulator of the mitochondrial stress response across species [92–96]. Of the four known the eIF2α kinases in mammals (GCN2, PERK, HRI and PKR), HRI has emerged as the main activator of the ISR upon acute mitochondrial dysfunction [95,96]. Interestingly, HRI is not conserved in Drosophila. Instead, we found that in Drosophila PERK senses perturbations in mitochondrial remodelling and likely acts to direct changes during stress and differentiation consistent with other reports [75,76,97]. Thus, PERK may represent a basal, conserved sensor of mitochondrial dysfunction across species.

Despite numerous reports linking mitochondrial dysfunction to the ISR, precisely how mitochondrial dysfunction and more specifically impairment of mitochondrial remodelling activates PERK remained unknown. Through a structure/function analysis we found that mutant PERK, unable to sense misfolded proteins, could still be activated by *Complex V* knockdown. This suggests that imbalances in the ER lipid bilayer composition activate PERK through its membrane domain in response to *Complex V* knockdown. Consistent with this, in *Complex V* knockdowns we observed a PERK-dependent increase in lipid droplets, which are thought to act as reservoirs to reduce ER lipid toxicity [98,99]. This increase in lipid droplets was also dependent on ATF4 suggesting that PERK promotes lipids droplets via ATF4.

Given our findings highlighting the importance of ER lipid bilayer stress in driving phenotypes caused by mitochondrial dysfunction, how disrupting mitochondrial remodelling induces ER lipid bilayer stress remains a critical avenue of future research. In the ER, lipid bilayer stress has been proposed to be caused by decreased phosphatidyl choline (PC) to phosphatidyl ethanolamine (PE) ratios [100,101], inositol depletion [102], increased lipid saturation [53,103], increased sterol levels [103,104], and increased protein-to-lipid ratios [54]. Which, if any, of these disruptions in mitochondrial remodelling causes remains to be determined; however decreased PC to PE ratios in whole Barth syndrome patient-derived cells and mouse organs deficient in tafazzin which are unable to remodel their mitochondria have been observed [105]. Thus, impairment of mitochondrial remodelling might perturb lipid exchange between mitochondria and the ER resulting in decreased ER PC to PE ratios, which could cause PERK activation. Interestingly, PERK has recently been shown to be enriched at mitochodria-ER contact sites [62]. Thus, PERK is well placed to act as sensor when lipid exchange between mitochondrial and the ER goes awry. Together, our data indicate that inhibiting mitochondrial remodelling induces lipid bilayer stress and activates PERK causing cell death and potentially contributing to disease.

The ISR is implicated in the etiology of several diseases linked to mitochondrial dysfunction including Alzheimer’s disease, Parkinson’s disease, Huntington disease, amyotrophic lateral sclerosis (ALS) and Charcot-Marie-Tooth disease [106]. Additionally, pharmacological or genetic inhibition of PERK has been shown to be neuroprotective in Drosophila models of Parkinson’s disease, and mouse models of frontotemporal dementia and Alzheimer’s disease [106]. Therefore, our findings provide important insights into how stem and germ cell function is potentially altered in individual suffering from these disorders. It also suggests that ER lipid bilayer stress may be a major driver of diseases phenotypes caused by mitochondrial dysfunction.

## Materials and methods

### *Drosophila* husbandry and genetics

All fly stocks were reared at 25°C with controlled humidity on standard medium (cornmeal, agar, yeast and molasses). All stocks used are listed in S1 Table. Genotypes for each figure are listed in S2 Table. All early germline *Complex Vα* and *Complex Ve* knockdowns were driven by either *UAS-Dcr2; nos-GAL4 (NGT40)* or *UAS-Dcr2;; nos-GAL4*::*VP16* at 25°C. Cyst knockdown was driven by *UAS-Dcr2;; bam-GAL4* at 29°C.

### Generation of PERK knockout lines

Flies ubiquitously expressing PERK gRNA (BDSC 77328) were crossed to flies expressing Cas9 (BDSC 67083) in the germline. 10–15 males with both Cas9 and PERK gRNA were crossed to a third chromosome balancer strain (TM3Sb/TM6B). From this cross, two knockout stocks were established using single males, one with a single base pair deletion (PERK^1^) and the other with 14 base pair deletion (PERK^2^).

### Generation of *PERK-ΔLD* line

We generated *PERK-ΔLD* line using scarless gene editing (flycrispr.org/scarless-gene-editing). All PCR amplification and plasmid assembly was performed using using Q5 High-Fidelity DNA Polymerase (NEB, M0491) and NEBuilder HiFi DNA Assembly Master Mix (NEB, E2621), respectively. The homology directed repair was performed by using a pCFD4d plasmid (a gift from Phillip Zamore; Addgene, 84005) expressing two gRNAs and a pScarlessHD-DsRed-w+ (a gift from Kate O’Connor-Giles; Addgene, 80801) homologous recombination (HR) plasmid lacking the PERK lumenal domain. The gRNAs (5’-GATCCCGCCCAGGTGCTAGC-3’; 5’-GTTATGCGCTTAATGGCGTAT-3’) targeting the gene region upstream and downstream of the lumenal domain were cloned into the pCFD4d plasmid. The PERK gene region was PCR amplified from genomic DNA of vas-Cas9 flies (BDSC 51324). The HR plasmid contained ~1kb of the PERK gene region upstream and downstream of the respective gRNA cut sites. A 3xHA tag sequence was added after the PERK ER signal peptide followed by 457 bp insertion of exon 2 directly upstream of TTAA site (gene region: 3R:complement [5460953..5461413], r6.43). Two silent mutations were made in the PERK sequence in the HR plasmid to prevent its cleavage by gRNA #2 bound Cas9. To facilitate screening, we inserted dsRed driven 3xP3 *Drosophila* eye promoter flanked by piggyBac (PBac) transposon ends for scarless removal.

pCFD4d and HR plasmids were injected into vas-Cas9 flies by Rainbow Transgenic Flies, Inc. The injected flies were balanced, and the progeny screened for dsRed expression. DsRed+ flies were established as a stock and the *PERK* gene was fully sequenced to verify deletion of the lumenal domain. The dsRed+ flies were crossed to a strain expressing the PBac transposase ubiquitously (BDSC 8285) and the F2 dsRed- flies were established as a stock following verification of scarless removal of dsRed and PBac transposons ends by sequencing.

### Generation of Complex II and V mutants

The following CRISPR gRNA was designed using flyCRISPR Target Finder (https://flycrispr.org/target-finder/): *Complex Vα*, 5’-GTCCGCCCGCCTGGCGTCCT-3’; *Complex Vg*, 5’-GTTTGGCTACCAAGGGATC-3’; and *SdhD*, 5’-GCCCTCTCGTTGCTTCTGCG-3’. Sense and antisense 5’ phosphorylated oligonucleotides with overhangs sequences complementary to the overhangs generated by BbsI were annealed and ligated into a BbsI cleaved pU6-BbsI-chiRNA plasmid. Plasmid DNA was injected by BestGene Inc. into a vas-Cas9 strain (BDSC 55821).

### Generation of *UASp-cyclophilin D*

Human *cyclophilin D* was PCR amplified from human HQB17 osteosarcoma cDNA using Phusion High-Fidelity DNA Polymerase (NEB, M0530) (See S3 Table for primers). Human *cyclophilin D* was inserted downstream of the Drosophila Hsp60A mitochondrial targeting sequence (amino acids 1 to 26) and upstream of a HA tag (YPYDVPDYA) between the NdeI and EcoRI sites of the pVALIUM22 vector using NEB Gibson Assembly (NEB, E2611S). The cyclophilin D pVALIUM22 vector was injected into y[1] sc[1] v[1]; P{y[+t7.7] = CaryP}attP2 embryos expressing PhiC31 integrase by BestGene Inc.

### Ovary immunofluorescence

Adult ovaries were stained according to standard procedures. Briefly, ovaries from well-fed or less than one day old flies were dissected in PBS and fixed in 4% formaldehyde (Thermo Scientific, 28908) in PBS for 15 min. For anti-phospho-eIF2α staining, ice-cold Dissection Buffer (10 mM Tris-HCl [BioShop Canada, TRS001] pH 6.8, 180 mM KCl [Sigma-Aldrich, P3911], 50 mM NaF [BioShop Canada, SFL001]) was used for dissection and fixation. Ovaries were then permeabilized with 1% Triton X-100 (BioShop Canada, TRX506) in PBS for up to 1 hour. Ovaries were incubated with primary antibodies diluted in 1% PBST (1% (w/v) bovine serum albumin [BSA, BioShop Canada, ALB001], 0.1% Triton X-100, PBS) overnight at 4°C followed by incubation with the appropriate secondary antibodies diluted in 1% PBST for 2 hours at room temperature. Ovaries were mounted in VECTASHIELD Antifade Mounting Medium (BioLynx, VECTH1000). All images were acquired with either a Leica SP8 inverted scanning confocal microscope using 10x (NA 0.4) and 63x (NA 1.4, immersion oil) objectives, Nikon Eclipse Ti inverted scanning confocal microscope using 10X (NA 0.3) objective and 63x (NA 1.4, immersion oil) objectives or a Zeiss LSM780 inverted scanning confocal microscope using 10X (NA 0.30) and 40X (NA 1.4, immersion oil) objectives. All experiments were performed using multiple sections (z-stacks) from confocal images. Image analysis and maximum projections was performed using FIJI [107]. All Complex Vα knockdown rescue experiments were immunostained to ensure the lack of Complex Vα protein expression.

The following primary antibodies were used: mouse anti-1B1 (1B1; 1:50) deposited to the DSHB by Lipshitz, H.D.; mouse anti-Cnx99a (Cnx99A 6-2-1; 1:1000) deposited to the DSHB by Munro, S.; rabbit anti-Vasa (1:5000; a gift from Ruth Lehmann); rabbit anti-Vasa (1:10 000; a gift from Prashanth Rangan); mouse anti-C(3)G (1:1000; a gift from R. Scott Hawley); mouse anti-ATP5a (1:1000; Abcam, ab14748); mouse anti-HA tag (1:1000; Abcam, ab130275); chicken anti-GFP (1:1000; Aves Labs, GFP-1010); rabbit anti-HA tag (1:1000; Cell Signaling Technology, 3724); rabbit anti-phospho-eIF2α (Ser51) (1:150, Cell Signaling Technology, 3597); rabbit anti-cleaved Drosophila Dcp-1 (Asp216) (1:100; Cell Signaling Technology, 9578). The following secondary antibodies were used at a 1:500 dilution: donkey anti-mouse Cy3 (Jackson ImmunoResearch Labs, 715-165-151); donkey anti-rabbit Cy3 (Jackson ImmunoResearch Labs, 711-165-152); donkey anti-rabbit DyLight 405 (Jackson ImmunoResearch Labs, 711-475-152); goat anti-rabbit Oregon Green 488 (ThermoFisher Scientific, O-11038); goat anti-mouse Alexa Fluor 488 (ThermoFisher Scientific, A-11001); anti-chicken v 488 (ThermoFisher Scientific, A-11039).

### S2R+ transfection and immunofluorescence

S2R+ cells were cultured in 10% fetal bovine serum (FBS; Gibco, 12483020) + Schneider’s Drosophila Medium (Gibco, 21720024). HA-PERK and HA-PERK^ΔLD^ were generated from cDNA and cloned in the pMT-Puro vector (a gift from David Sabatini; Addgene, 17923) using using Q5 High-Fidelity DNA Polymerase (NEB, M0491) and NEBuilder HiFi DNA Assembly Master Mix (NEB, E2621). S2R+ cells were transfected with HA-PERK or HA-PERK^ΔLD^ using TransIT-Insect Transfection Reagent (Mirus Bio, MIR 6104) according to manufacturer’s protocol. Briefly, 1.6 x 10^5^ cells/mL were seeded in a well of a 6-well plate on the day of transfection. 2.5 μg of plasmids were mixed in 250 μL of Schneider’s Drosophila Medium with 5 μL of TransIT-Insect reagent. After an incubation of 15 min at room temperature, TransIT-Insect reagent:DNA complexes were added dropwise to the well. After 48 hours, 200 μM of copper sulfate (BioShop, CUS803.500) was added to cells. After 24 hours, cells were transferred to an eight-well chamber slide (Lab-Tek II, 154534) pre-coated with 0.1% poly-D-lysine (Sigma-Aldrich, P6407-5MG).

Cells were then fixed with 4% formaldehyde (Thermo Scientific, 28908) in PBS for 10 min, permeabilized in 0.1% (v/v) Triton X-100 (BioShop Canada, TRX506) in PBS for 15 min and incubated overnight at 4°C with primary antibody (see above). The next day, cells were incubated with secondary antibody for 2h at room temperature and mounted in VECTASHIELD Antifade Mounting Medium (BioLynx, VECTH1000). All images were acquired with a Leica SP8 inverted scanning confocal microscope using a 63x (NA 1.4, immersion oil) objective. The data in S2 Fig representative of approximately 100 cells assessed from three fields of two independent experiments.

### Membrane potential imaging

Prior to imaging, freshly dissected ovaries were incubated in Schneider’s Drosophila Medium (Gibco, 21720024) containing 10 nM tetramethylrhodamine, methyl ester (TMRM) (Invitrogen, T668) and 1 μM sodium tetraphenylborate (TPB) (Sigma, T25402) for 30 min in 8-well Lab-Tek II Chambered Coverglass, 1.5 borosilicate glass chambers. The 30-min incubation was essential for the equilibration of TMRM throughout the germaria. TMRM was imaged using a Zeiss LSM780 inverted scanning confocal microscope with a 40X (NA 1.4, immersion oil) objective. In some instances, samples were additionally incubated with 2 μg/ml oligomycin (Sigma, O4876) and 3 μM antimycin A (Sigma, A8674) for 5 min prior to re-imaging.

### *In vivo* global protein synthesis imaging and quantification

Protein synthesis was detected using Click-&-Go Plus 555 OPP Protein Synthesis Assay Kit (Click Chemistry Tools, 1494). Less than one day old ovaries were dissected in Schneider’s Drosophila Medium (Gibco, 21720024) and incubated with 50 μM of OPP reagent for 5 min with gentle rotation. Samples were washed twice with fresh Schneider’s Drosophila Medium followed by fixation in 4% formaldehyde (Thermo Scientific, 28908) in PBS for 20 min. Samples were washed twice in PBS + 3%(w/v) BSA (BioShop Canada, ALB001) for 5 min each and permeabilized in 0.5% Triton X-100 (BioShop Canada, TRX506) in PBS for 20 min. After two washes with PBS + 3% BSA, Click-iT reaction was carried out in the dark for 30 min with the Click-iT reaction cocktail. Samples were washed with Click-iT wash buffer and PBS + 1% BSA + 0.1% Triton X-100. Immunostaining was carried out according to standard procedure and appropriate antibodies. All images were acquired with a Leica SP8 inverted scanning confocal microscope using 63x (NA 1.4, immersion oil) objective.

OP-Puro fluorescence intensity was quantified using FIJI. We determined mean fluorescence intensity in two independent cytoplasmic regions of 0.7 μm x 0.7 μm in maximum projections of 3 slices. For each germaria, GSC fluorescence intensity was normalized to the mean fluorescence intensity of two non-adjacent follicle cells. Each experiment was performed in triplicate and germaria from over 10 ovary pairs were analyzed. Unpaired t-test was performed using (GraphPad, v9).

### Hydrogen peroxide imaging

Prior to imaging, freshly dissected ovaries were incubated in Schneider’s Drosophila Medium (Gibco, 21720024) containing 20 μM PO1 in the absence or presence of 100 μM hydrogen peroxide for 30 min in 8-well Lab-Tek II Chambered Coverglass, 1.5 borosilicate glass chambers. PO1 was imaged using a Zeiss LSM780 inverted scanning confocal microscope with a 40X (NA 1.4, immersion oil) objective.

### Electron microscopy

Drosophila ovaries were dissected in PBS and fixed in 2.5% glutaraldehyde (Electron Microscopy Sciences (EMS) 16220) and 2% paraformaldehyde (EMS, 15710) in 0.1 M phosphate buffer (pH 7.4) at room temperature for 1 hour, and then overnight at 4°C. Ovaries were post-fixed with 1% osmium tetroxide (EMS, 19150) for 1 hour at 4°C, then stained en bloc with 1% uranyl acetate (EMS, 22400) in double-distilled H2O at 4°C for 1 hour. Dehydration series were carried out at 4°C using ethanol from 30% and 50% to 70%, then room temperature at 85%. To preserve mitochondrial crista structure, dehydration steps were limited to 5 min each. Ovaries were processed in a standard manner and embedded in Araldite 502 (Ted Pella, 18060; ref. 40). 500 nm semi-thin sections were stained with 0.1% toluidine blue (EMS, 22050) to evaluate the area of interest. 60 nm ultrathin sections were cut, mounted on formvar coated slotted copper grids and stained with uranyl acetate and lead citrate by standard methods. Stained grids were examined under a Philips CM-12 electron microscope (FEI) and photographed with a Gatan (4k × 2.7k) digital camera. Electron micrographs in S2B and S2C Fig are representative of at least three germaria analysed per genotype with at least three sections viewed for each.

### Clonal mosaic analysis

Clones were induced by heat shocking adult flies at 37°C in the morning and evening for 2 hours each for two consecutive days. Ovaries were dissected and analyzed 6, 11 and 14 days after the heat shock.

### Quantification of germline differentiation stages

For germarial stage specific quantification, germaria stained with anti-Vasa and anti-1B1 were carefully scored based on spectrosome/fusome morphology of the most mature stage present. For example, if only GSCs and 4-cell cysts were visible in a germarium, that germarium was scored as differentiated up to 4-cell cyst stage.

For ovary/ovariole differentiation stage quantification, germaria only pertains to the absence of any egg chambers, early stage pertains to stage 1–6 egg chambers, and late stage pertains to egg chambers from stage 7 and above.

### Quantification of cleaved Dcp-1

Two to three-day old well-fed ovaries were dissected as above and stained with rabbit anti-cleaved Dcp-1 and mouse anti-1B1. Under high magnification (63x) germaria were scored as cleaved Dcp-1 positive if they possessed strong signal near visible GSCs or cysts.

### Lipid droplet imaging and quantification

Two to three-day old ovaries were dissected in PBS and fixed with 4% formaldehyde in PBS for 15 min. After primary and secondary antibody incubation, ovaries were stained with BODIPY 493/503 (1 μg/mL; ThermoFisher Scientific, D3922) and Alexa Fluor 647 phalloidin (264 nM; ThermoFisher Scientific, A22287) in PBS for 15 min. Ovaries were rinsed twice with PBS and mounted in Fluoromount-G (ThermoFisher Scientific, 00-4958-02).

Ovaries were imaged on a Leica SP8 inverted scanning confocal microscope using a z-slice of 0.3 μm. Germaria image stacks were three-dimensionally reconstructed using Imaris software (Oxford Instruments, v9.8). A region of interest was drawn around each GSC identified by an anterior localization and presence of the spectrosome using manual contouring. The lipid droplets were detected using the surface tool to determine the number and volume of each lipid droplet in each GSC. The exported data was plotted using Prism (GraphPad, v9).

### Embryo and ovary RNA extraction, and RT–qPCR

Thirty to forty females expressing maternal-tubulin-GAL4 and a UAS-RNAi were crossed to wildtype males and maintained on apple juice plates (agar, apple juice, Nipagin, sucrose) with yeast paste. After synchronization of egg lay, embryos were collected for two hours and dechorionated using liquid bleach. Total RNA was extracted from embryos using Tri-Reagent (BioShop Canada, Cat#TRI118), chloroform (Sigma-Aldrich, 472476), 2-propanol (Sigma-Aldrich, I9516) and ethyl alcohol (Commercial Alcohols, 22734). Purified RNA was treated with Turbo DNAse (ThermoFisher Scientific, 2238G2). For cDNA synthesis, 2000 ng of RNA was used with SuperScript II Reverse Transcriptase kit (ThermoFisher Scientific, 18064014) and oligo dT-20mer (Integrated DNA Technology, 51-01-15-01).

For *Atf3* mRNA expression in the ovary, 20–40 less than 1-day old ovaries were dissected, and total RNA was extracted using Tri-Reagent as above. For cDNA synthesis, 500 ng of RNA was used with SuperScript IV Reverse Transcriptase (ThermoFisher Scientific, 18090050) and oligo dT-20mer.

Quantitative PCRs were carried out on 1/50 of reverse transcription reaction and 300 nM of each primer pair using the SensiFAST SYBR No-ROX kit (FroggaBio, BIO-98050) and a Bio-Rad CFX384/C1000 Touch system (Bio-Rad). The PCR program was as follows: 2 min at 95°C; 45 cycles of 95°C for 5 s and 60°C for 30 s. Results were normalized to the mean of value obtained of CG8187, CG2698 and Und. Gene knockdown was normalized to the relative expression level in *mCherry* knockdown embryos. Results were calculated using the following formula: ΔΔCt = 2^-(ΔCtRNAi− ΔCt_mCherry RNAi_), where ΔCt = Ct (gene)–Ct (mean of CG8187, CG2698 and Und). Primer pairs are listed in S3 Table. The expression data in S6J and S8B Figs are the means of three technical replicates.

### Eggs laying and hatching

Four one week old females of the respective genotypes and two w1118 males were maintained on apple juice plates with yeast paste. Fresh apple juice plates with yeast plates were exchanged every 24 hours for 3 days. The number of eggs laid were counted and averaged over the three days. For hatching, hatched eggs on apple juice plates were counted 48 hours after egg lays. The data shown in S8 Fig are two independent replicates.

### Statistics and reproducibility

All experiments were repeated at least three individual times using at minimum ten females overall. For representative images and quantifications in Figs 1B–1E, 2H, 3A, 3B, 3D–3I, 4, 5, 6, S3, S4D–S4H, S6B–S6J. S8, S12A, S12B, S12D and S12E, fly progeny were generated with at least two independent crosses. For targeted suppressor screens and experiments in S6A, S8A and S12C Figs, n > 10 ovary pairs were analyzed.

Graphs and statistics in relevant figures were generated using Prism 9.2 (GraphPad) and underlying data is provided (S4 Table). For lipid droplet number and total volume per GSCs (Figs 6I–6J and S11D–S11E), outliers were detected and removed using the ROUT method with Q = 0.1%. The data without outliers was normalized to the mean of control and plotted as mean ± s.d. and statistical significance was assayed using Brown-Forsythe ANOVA followed by a Games-Howell post hoc test. For ovary (S6A, S8A and S12C Figs), ovariole (S2D Fig) and germaria (Figs 1E, 4H, 5E, 6C, S3D and S4H) differentiation stage quantification, all replicates were pooled and the percentage for each stage in each genotype was displayed. For the frequency of germline mosaic clones (Figs 1I–1J and S5G–S5I), cleaved Dcp-1 (Fig 6D), phospho-eIF2α (Figs 3F and S7C and S7F) and 4E-BP reporter (Fig 3I) per GSC or germaria, results were analyzed as individual 2x2 contingency tables where each value is an exact count, each row defines the genotype, and each column defines the outcome. The data was graphed as a frequency. Statistical significance was assayed using a two-sided Fisher’s exact test. For *Atf3* mRNA fold change, data were plotted as mean ± s.d. and an unpaired t-test with Welch’s correction was performed. For the frequency of meiotic 8-cell egg chambers (Fig 6G), each data point represents the percentage of meiotic 8-cell egg chambers per replicate and were plotted as mean ± s.d. Statistical significance was assayed using one-way ANOVA followed by a Tukey post hoc test.

## Supporting information

S1 Fig Knockdown of *Complex V* or *Phb2* impairs mitochondrial cristae. Representative electron micrographs of Control (mCherry) **(a)**, *Complex III subunit RFeSP* **(b)**, *Complex IV subunit Va* **(c)**, *Complex Vα* **(d)**, and *Phb2* **(e)** KD germ cells. RNAi were driven by *nos-GAL4*. Scale bars represent 250 nm. For exact genotypes see S2 Table.
(TIF)

S2 Fig Mitochondrial cristae remodelling factors Opa1, and Phb1 and 2 are essential for germline differentiation. Representative germarium images of *Phb1* **(a)** and *Opa1* ***(c)*** KDs driven by *nos-GAL4*. Scale bars represent 10 μm. **(c)** Phenotypic quantification of germline differentiation in whole ovaries of the indicated RNAi lines driven by *nos-GAL4*. Number of ovaries analyzed for each genotype is indicated at the top. For exact genotypes see S2 Table.
(TIF)

S3 Fig Complex V plays a non-essential role in germline stem cell proliferation. **(a-c)** Representative images of 2–3 days old *CVα* KD, *bam*^*–/+*^ **(a)**, *CVα* KD, *bam*^*–/–*^**(b)**, and *bam*^*–/–*^**(c)** germaria (GSCs; white-dashed line). Scale bars represent 10 μm. **(d)** Quantification of latest germline differentiation stage in germaria of indicated genotypes represented as proportion. Number of germaria scored is indicated above each bar. **(e)** Quantification of GSCs per germarium of the indicated genotypes (*n* = 193 germaria for *CVα* KD, *bam*^*–/+*^; *n* = 125 germaria for *CVα* KD, *bam*^*–/–*^; *n* = 100 for control *bam*^*–/–*^). Data are the mean ± s.d. Statistical analysis was performed using one-way ANOVA followed by Games-Howell multiple comparison’s test. For all KDs, RNAi were driven by *nos-GAL4*. For exact genotypes see S2 Table.
(TIF)

S4 Fig Activation of the permeability transition pore does not inhibit germline stem cell differentiation. **(a)** Models of the opening of the mitochondrial permeability transition pore (PTP) by the binding of human cyclophilin-D (CypD) to Complex V (CV). **(b, c)** Representative electron micrographs of Control **(b)** and human CypD-HA expressing **(c)** germline mitochondria. Arrows indicate swollen mitochondria characteristic of the opening of the PTP. Representative confocal images of whole ovary **(d, e)** and germaria **(f, g)** expressing Control (*UAS-GFP*) **(d, f)** or human CypD-HA **(e, g)**. **(h)** Quantification of latest differentiation stage in germaria of **(f, g)**. **(i)** Representative images of 2–3 day old *CVc* KD germaria. White-dashed line indicates the germline. All overexpression and knockdown constructs were driven by *nos-GAL4*. For exact genotypes see S2 Table.
(TIF)

S5 Fig Clonal analysis of electron transport chain mutants. **(a)** Schematic representation of oxidative phosphorylation. Complexes I, III and IV pump protons out of the mitochondrial matrix to generate a proton gradient and a membrane potential. Complex V uses the proton gradient to generate ATP. Antimycin (AA) inhibits Complex III and oligomycin (oligo) inhibits the ATP synthesis role of Complex V. **(b)** Graphical representation of *Complex II subunit* D, *Complex III Cyt-c1* and *Complex IV subunit 5A* loss-of-function mutations. **(c-f)** Representative images of Control **(c)**, Control + AA + oligo **(d)**, *Complex III Cyt-c1* **(e)**, and *Complex IV subunit 5A* **(f)** mosaic ovaries 16-days post-clone induction incubated with tetramethylrhodamine, methyl ester (TMRM) to visualize the mitochondrial membrane potential (ΔΨ; magenta) live. Mutant germ cells do not express GFP (blue) and are outlined in white-dashed lines. Scale bars represent 10 μm. **(g-i)** Frequency of 5-, 11- or 14-days after clone induction of *Complex II subunit D* **(g)**, *Complex III Cyt-c1* **(h)**, and *Complex IV subunit 5A* **(i)** mosaic ovaries. Undifferentiated: germline cells in Region 1 including GSC, cystoblasts, 2-, 4- and 8-cell cysts. Differentiated: germline cells in Region 2. Number of germaria observed are given inside the bars. P-values were calculated using Fisher’s exact test. For exact genotypes see S2 Table.
(TIF)

S6 Fig Knockdown of ATF4 restores germ cell differentiation. **(a)** Double knockdown of *CV*, and knockdown or overexpression of indicated mito-nuclear retrograde signalling genes. Number of ovaries analyzed is indicated at the above the bar. Ovaries were 1–7 days old. **(b-e)** Representative images of 2–3 day old *CVα* KD **(b)**, *CVα*, *foxo* KD **(c)**, *CVα* KD, *foxo*^*Δ94*^ mutant **(d)**, and *CVα*, *Ire1* KD **(e)** germaria. Images shown are representative of at least 100 germaria. **(f, g)** Representative images of 2–3 days old Control **(f)** and *CVα* KD **(g)** ovaries expressing FOXO activity reporter (magenta), *thor4p-*dsRed. **(h, i)** Representative images of 2–3 days old Control **(h)** and *CVα* KD **(i)** ovaries expressing JNK activity reporter (gray), *TRE-*dsRed. For **(f-i)** at least 30 ovarioles across five ovary pairs were analyzed. White-dashed line indicates the GSCs. For all confocal images, scale bars represent 10 μm. **(j)** *Atf3* mRNA levels in Control (*mCherry*) KD and *CVα* KD ovaries expressing P35 to increase the number of germ cells. Data are the mean ± s.d. and statistical significance was calculated using unpaired t-test with Welch’s correction. All RNAi were driven by *nos-GAL4*. For exact genotypes see S2 Table.
(TIF)

S7 Fig Loss of mitochondrial remodelling factors *Opa1* and *Phb1* activate the integrated stress response. **(a, b)** Representative images of 2–3 day old Control (*mCherry*) **(a)**, and *Opa1* **(b)** KD germaria. **(c)** Frequency of GSCs with high phosphorylated eIF2α relative to surrounding somatic cells from **(a, b)**. **(d, e)** Representative images of 2–3 day old Control (*mCherry*) **(f)** and *Phb1* **(e)** KD germaria. **(f)** Frequency of GSCs with high phosphorylated eIF2α relative to surrounding somatic cells from **(d, e)**. The number of GSC analyzed and P-value (Fisher’s exact test) are given above bars. For all confocal images, scale bars represent 10 μm and white-dashed lines demark the GSCs. All RNAi were driven by *nos-GAL4*. For exact genotypes see S2 Table.
(TIF)

S8 Fig PERK, but not GCN2, knockdown rescues germ cell differentiation defects caused by Complex V knockdown. **(a)** Frequency of phenotype of Control (*CVα* KD only), *GCN2* and *PERK* double *CVα* KDs driven by *nos-GAL4*. Number of ovaries analyzed are indicated at the top. Ovaries were 2–3 days old. **(b)** Knockdown efficacy of *GCN2* and *PERK* RNAi lines used. RNAi were driven by *maternal-tubulin-GAL4* and maternally deposited mRNA levels were assessed in less than 2 hour old embryos. RNA levels were normalized to Control (*mCherry*) RNAi. **(c-e)** Representative images of 2–3 days old *CVα* KD **(c)**, *CVα*, *GCN2* KD **(d)**, and *CVα*, *PERK* KD **(e)** ovaries. Scale bars represent 100 μm. **(f)** *PERK* knockout alleles with 1 bp (*PERK*^*1*^) and 14 bp (*PERK*^*2*^) deletion are predicted to generate truncated and non-functional protein products. **(g)** Number of eggs laid per female of the Control (*w*^*1118*^) and *PERK* transheterozygous mutants (*PERK*^*1*^*/PERK*^*2*^). **(h)** Percent of hatched eggs laid by females in **(h)**. Two replicates were performed for **(g, h)**. For exact genotypes see S2 Table.
(TIF)

S9 Fig Generation and characterization of PERK lumenal domain deletion mutants. **(a)** Schematic of the generation of PERK lumenal domain deletion mutants using CRISPR/Cas9 and homologous recombination. Two guide RNAs were expressed flanking regions the PERK lumenal domain (exons 1–3). A plasmid containing the lumenal domain deletion and the piggyBac inverted repeat flanked 3xP3 driven dsRed was used to drive homologous recombination. After expression of piggyBac transposase, the dsRed region was excised scarlessly. **(b)** Graphical schematic of Drosophila wild-type and *PERK*^*ΔLD*^ domains. **(c, d)** Representative images of 2–3 day old homozygous *PERK*^*ΔLD*^ **(c)** and unedited control (*w*^*1118*^) **(d)** germaria. Scale bars represent 10 μm. **(e-g)** Representative images of untransfected S2R+ cells **(e)**, and S2R+ transfected with *HA-PERK* **(f)** and *HA-PERK*^*ΔLD*^ **(g)**. Scale bars represent 5 μm. For exact genotypes see S2 Table.
(TIF)

S10 Fig Germ cell-specific loss or reduction of Complex V does not cause oxidative stress. **(a-b)** No increase in hydrogen peroxide was observed in mutant *CVg* mosaic ovaries. Live representative image of *CVg*^*–/–*^mosaic ovaries 12-days post-clone induction incubated with the hydrogen peroxide sensor, peroxy orange-1 [110] (20 μM) in Schneider’s medium in the absence **(a)** or presence **(b)** of hydrogen peroxide (100 μM). GFP-negative cells marked by the white-dashed line represent *CVg* null mutant cells. **(c-e)** No increase in oxidative stress was observed *CVa* or *CVc* germline (white-dashed line) specific KD ovaries. Representative images of fixed ovaries Control **(c)**, *CVα* **(d)** and *CVc* **(e)** germline KDs. Oxidative stress was assessed using the *GstD1-GFP* reporter [63]. Ovaries were fixed and stained with anti-Vasa to mark the germline and anti-GFP. For exact genotypes see S2 Table.
(TIF)

S11 Fig ATF4 induces the formation of lipid droplets in response to mitochondrial remodelling failure. **(a-c)** Representative images of 2–3 day old Control **(a)**, *CVα* KD **(b)**, and *CVα* KD, *ATF4* KD **(c)** GSCs (white-dashed line). BODIPY 493/503 marks lipid droplets. Scale bars represent 5 μm. **(d)** Quantification of number of lipid droplet per GSC normalized to mean of control for the indicated genotypes (*n* = 45 for Ctrl; *n* = 49 for *CVα* KD; and *n* = 47 for *CVα* KD, *ATF4* KD). **(e)** Quantification of total lipid droplet volume per GSC normalized to mean of control for the indicated genotypes (*n* = 45 for Ctrl; *n* = 47 for *CVα* KD; and *n* = 47 for *CVα* KD, *ATF4* KD). All RNAi were driven by *nos-GAL4*. For exact genotypes see S2 Table.
(TIF)

S12 Fig Mitochondrial remodelling failure induces Hid-Dronc/Dark mediated cyst death and precocious meiosis. **(a, b)** Representative images of 1 day old *CVα* KD **(a)** and *CVα* KD, P*35* overexpression **(b)** ovaries. Scale bars represent 50 μm. **(c)** Cell death suppressor screen in *CVα* KD background. Number of ovaries analyzed are indicated at the top. Ovaries were 1–4 days old. **(e, f)** Confocal image slices for **(e)** Fig 6D: Control and **(f)** Fig 6E: *CVα* KD with *P35* overexpression germaria driven by *nos-GAL4*. White dashed lines outline the Region 3 egg chamber and numbers indicate cyst nuclei. Scale bars represent 5 μm. For exact genotypes see S2 Table.
(TIF)

S13 Fig Inner mitochondrial membrane remodelling maintains ER lipid homeostasis during GSC differentiation. Schematic showing that the inability to remodel the inner mitochondrial membrane induces ER lipid membrane stress and cell death. ER bilayer stress is induced upon the loss of Complex V and cristae formation. This stress is detected by PERK which then activates the ISR through the phosphorylation of eIF2α and induction of ATF4. Increased ISR activity increases the formation of lipid droplets potentially to alleviate ER bilayer stress.
(TIF)

S1 Table List of Drosophila strains used. (XLSX)

S2 Table List of all Drosophila genotypes used for figures. (XLSX)

S3 Table List of quantitative PCR primers. (XLSX)

S4 Table Data and statistics for all graphs. (XLSX)
