# Supplementary figures and images for: Mitochondrial remodelling is essential for female germ cell differentiation and survival
Source: PLoS Genet. 2023 Jan 25;19(1):e1010610. doi: 10.1371/journal.pgen.1010610 (PMC9901744; doi:10.1371/journal.pgen.1010610)

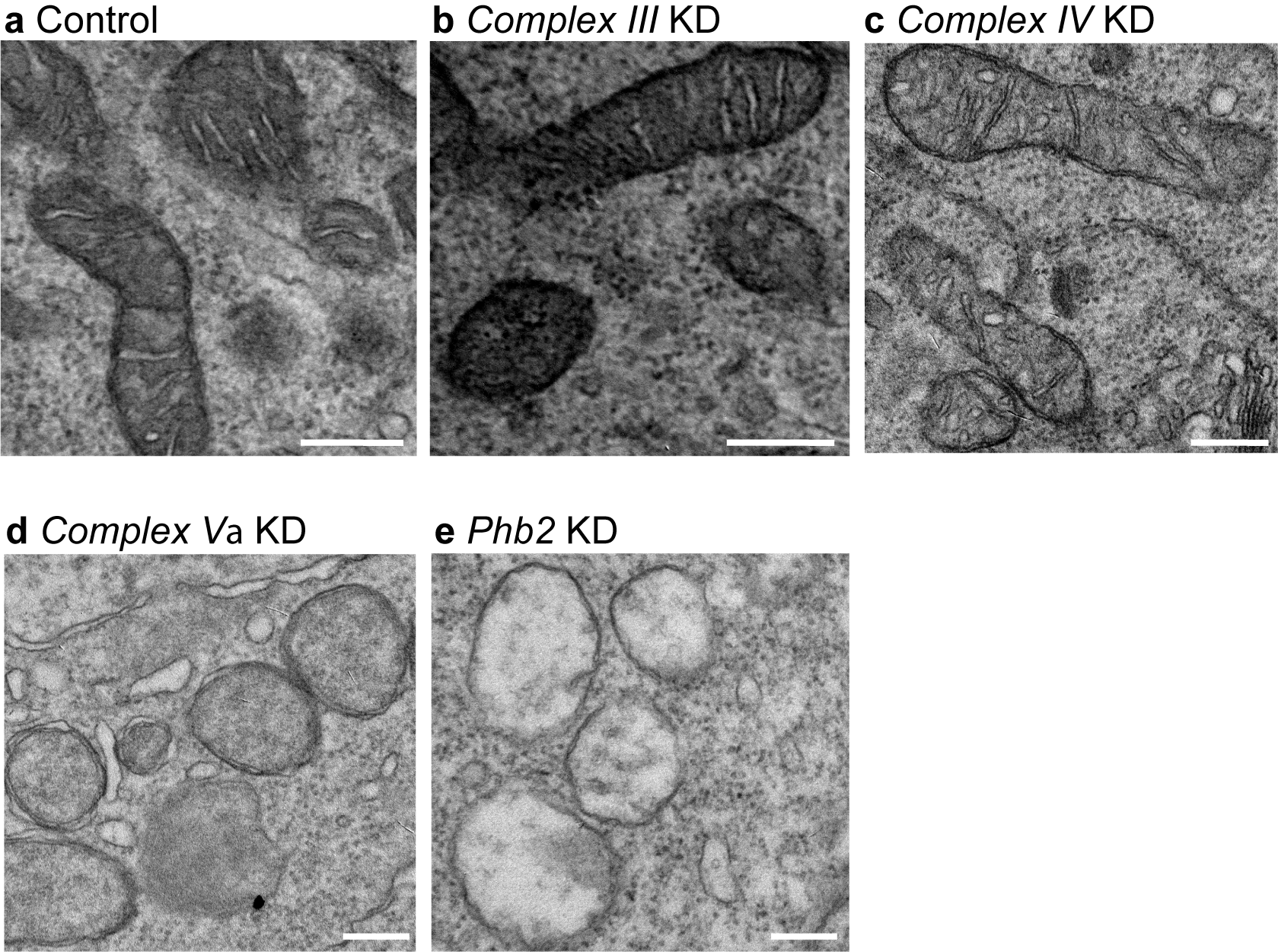

Supplement: S1 Fig — Representative electron micrographs of Control (mCherry) (a), Complex III subunit RFeSP (b), Complex IV subunit Va (c), Complex Vα (d), and Phb2 (e) KD germ cells. RNAi were driven by nos-GAL4. Scale bars represent 250 nm. For exact genotypes see S2 Table. (TIF) [file pgen.1010610.s001.tif]

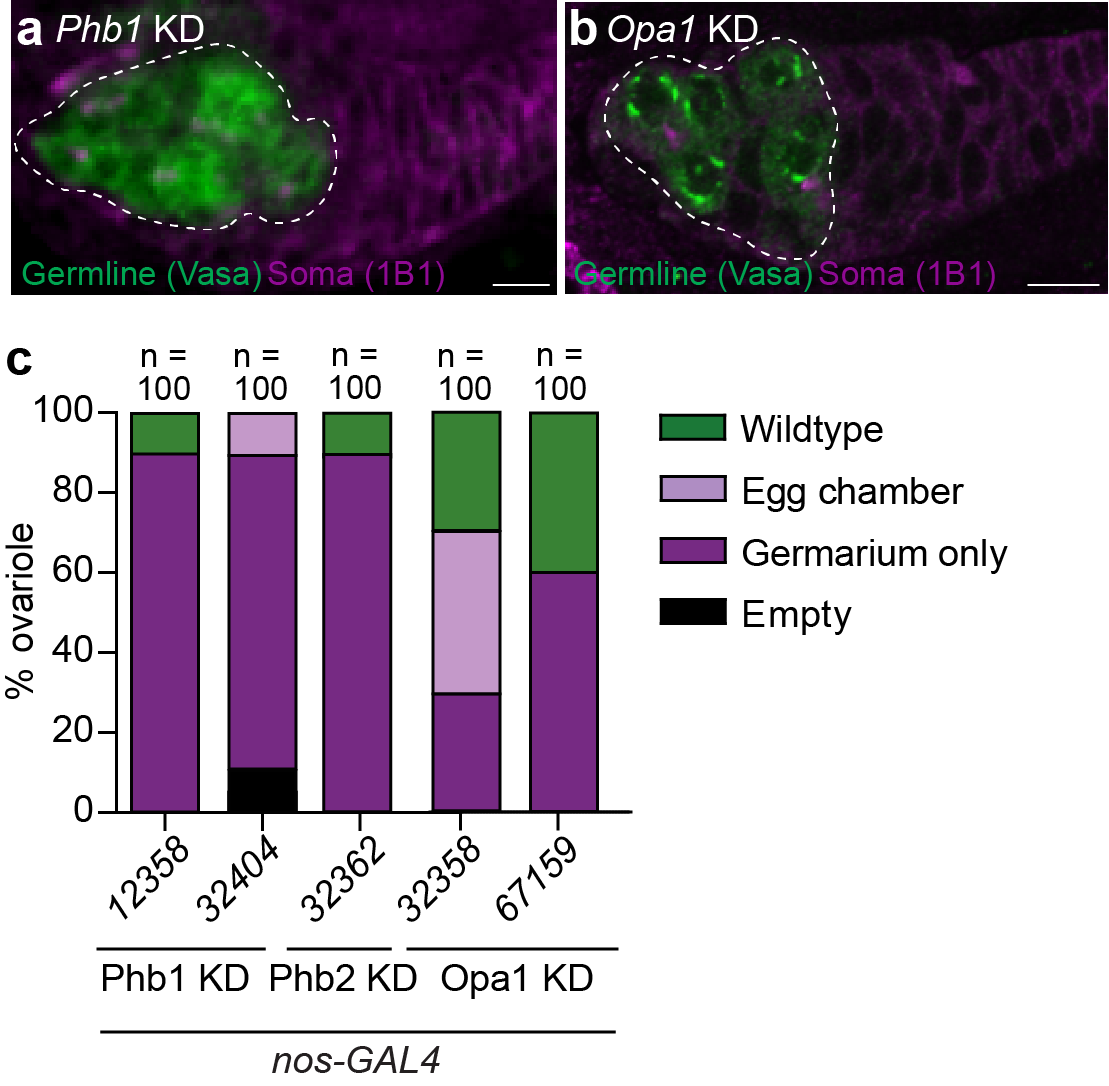

Supplement: S2 Fig — Representative germarium images of Phb1 (a) and Opa1 (c) KDs driven by nos-GAL4. Scale bars represent 10 μm. (c) Phenotypic quantification of germline differentiation in whole ovaries of the indicated RNAi lines driven by nos-GAL4. Number of ovaries analyzed for each genotype is indicated at the top. For exact genotypes see S2 Table. (TIF) [file pgen.1010610.s002.tif]

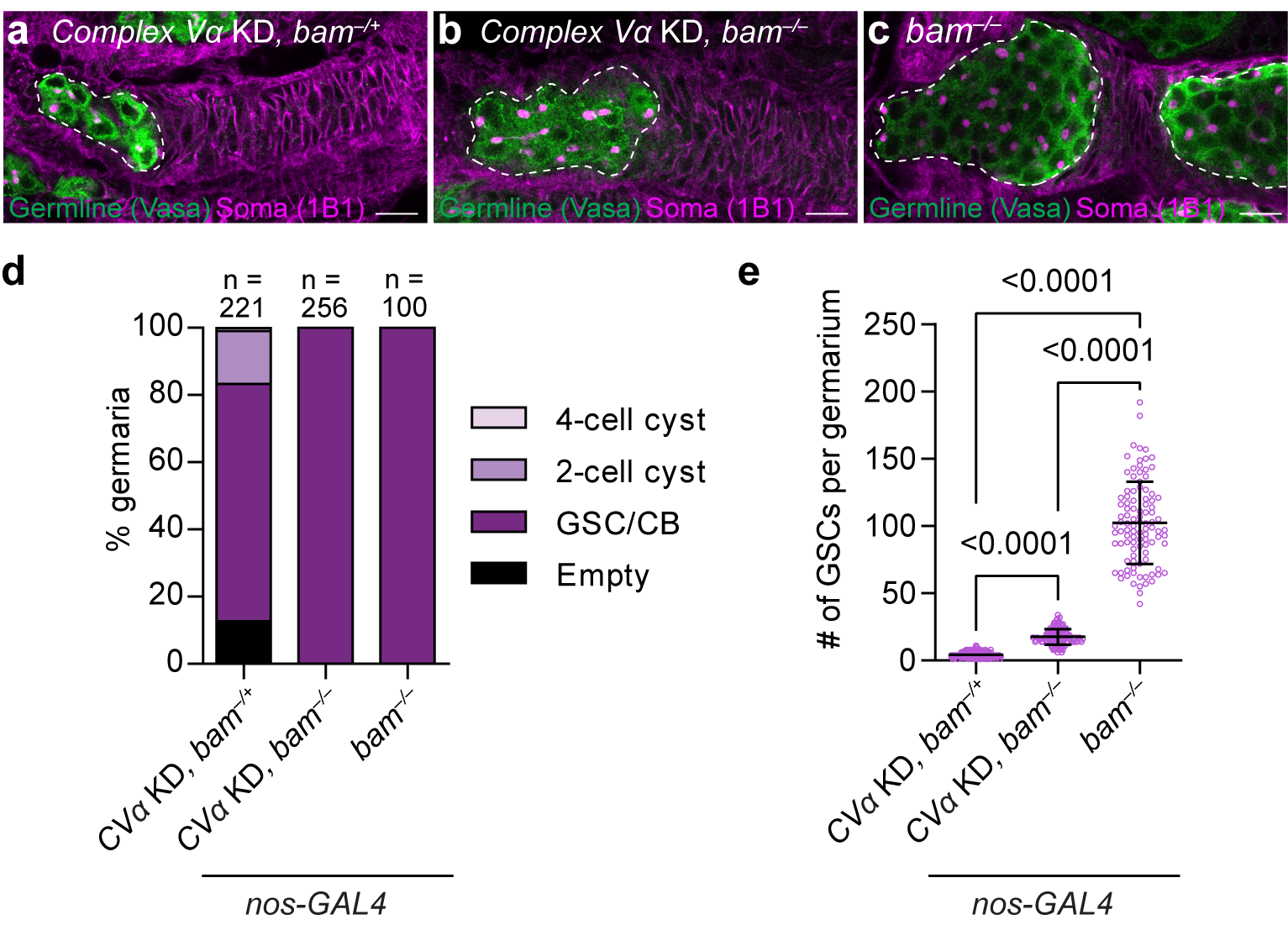

Supplement: S3 Fig — (a-c) Representative images of 2–3 days old CVα KD, bam–/+ (a), CVα KD, bam–/–(b), and bam–/–(c) germaria (GSCs; white-dashed line). Scale bars represent 10 μm. (d) Quantification of latest germline differentiation stage in germaria of indicated genotypes represented as proportion. Number of germaria scored is indicated above each bar. (e) Quantification of GSCs per germarium of the indicated genotypes (n = 193 germaria for CVα KD, bam–/+; n = 125 germaria for CVα KD, bam–/–; n = 100 for control bam–/–). Data are the mean ± s.d. Statistical analysis was performed using one-way ANOVA followed by Games-Howell multiple comparison’s test. For all KDs, RNAi were driven by nos-GAL4. For exact genotypes see S2 Table. (TIF) [file pgen.1010610.s003.tif]

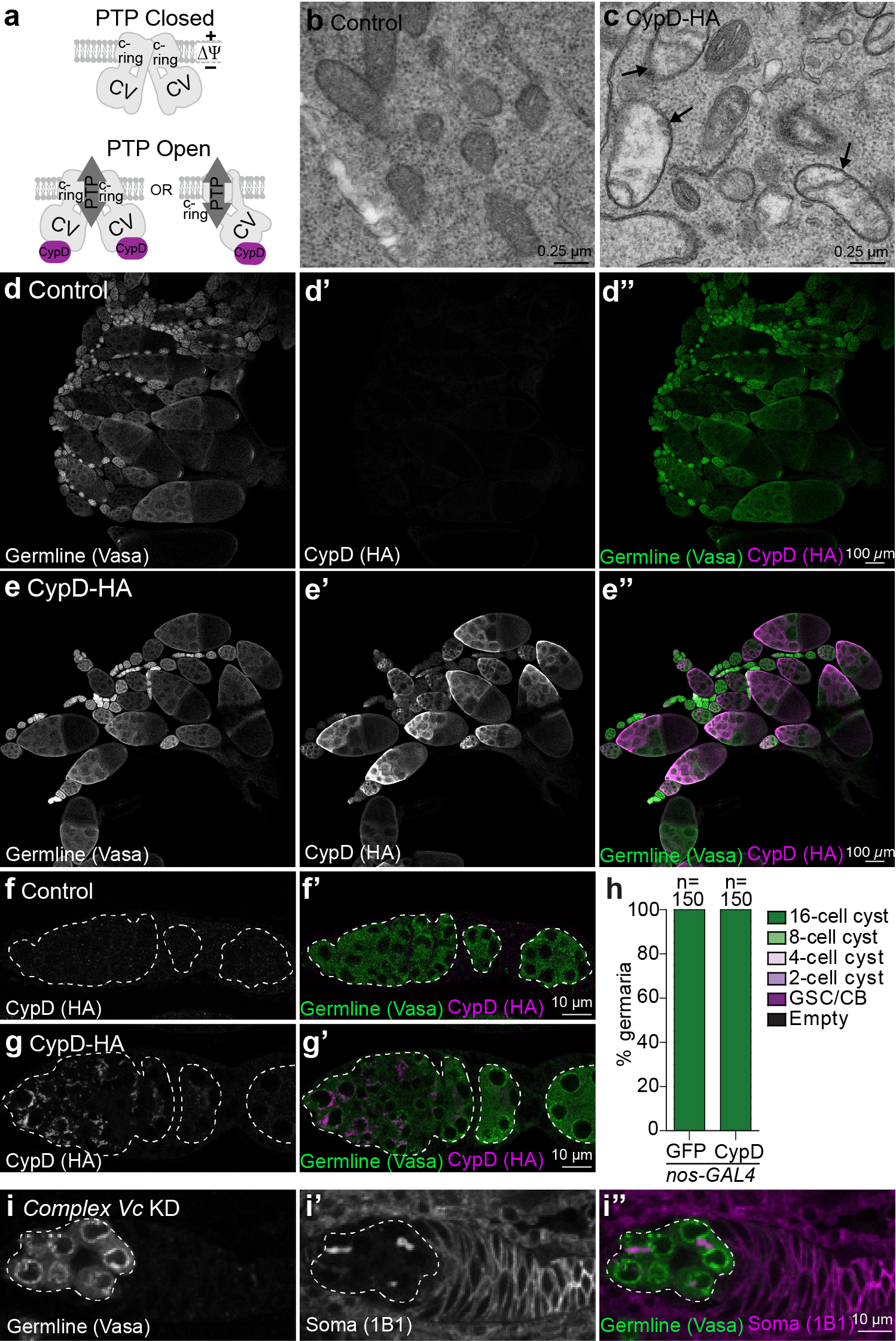

Supplement: S4 Fig — (a) Models of the opening of the mitochondrial permeability transition pore (PTP) by the binding of human cyclophilin-D (CypD) to Complex V (CV). (b, c) Representative electron micrographs of Control (b) and human CypD-HA expressing (c) germline mitochondria. Arrows indicate swollen mitochondria characteristic of the opening of the PTP. Representative confocal images of whole ovary (d, e) and germaria (f, g) expressing Control (UAS-GFP) (d, f) or human CypD-HA (e, g). (h) Quantification of latest differentiation stage in germaria of (f, g). (i) Representative images of 2–3 day old CVc KD germaria. White-dashed line indicates the germline. All overexpression and knockdown constructs were driven by nos-GAL4. For exact genotypes see S2 Table. (TIF) [file pgen.1010610.s004.tif]

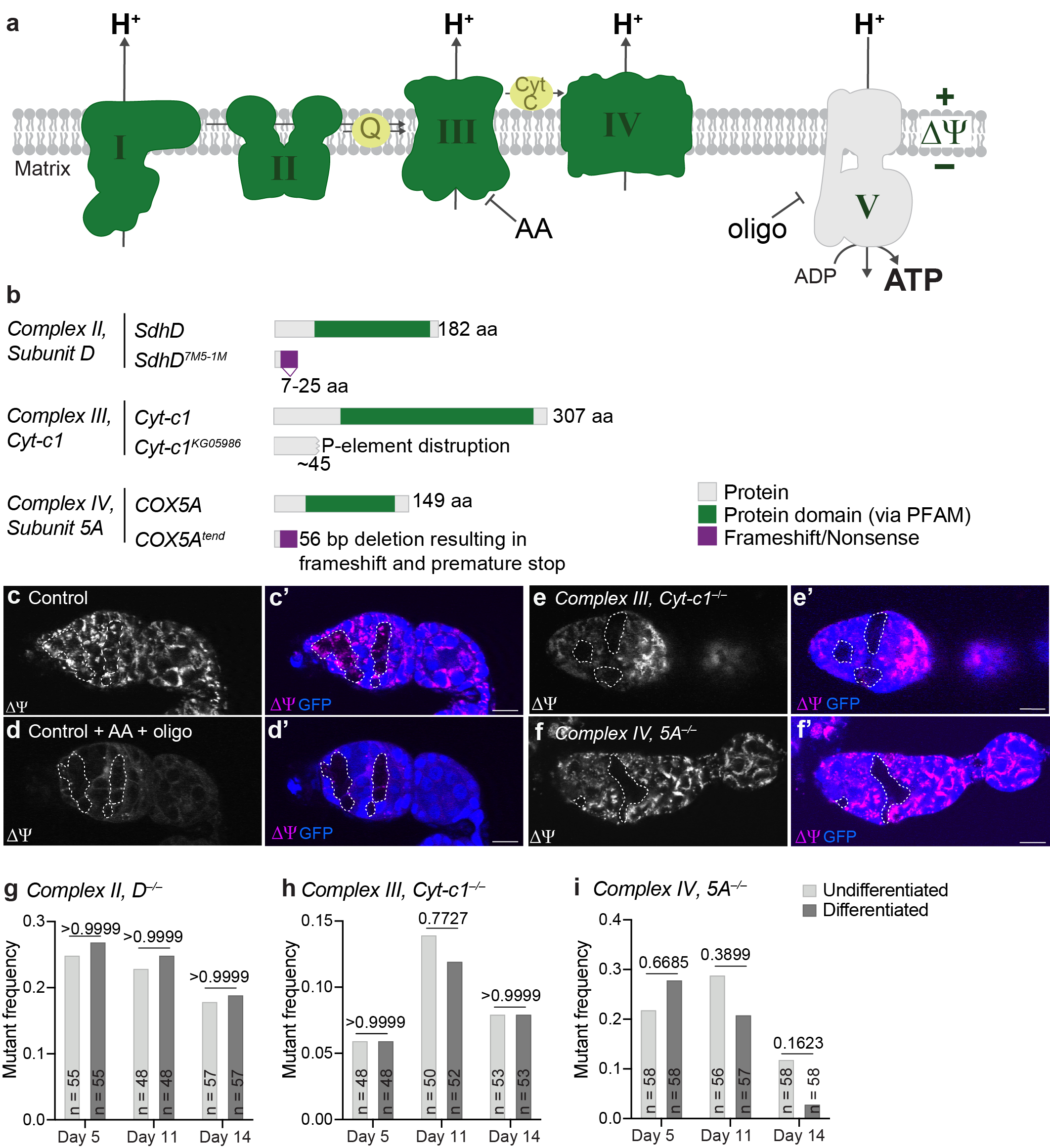

Supplement: S5 Fig — (a) Schematic representation of oxidative phosphorylation. Complexes I, III and IV pump protons out of the mitochondrial matrix to generate a proton gradient and a membrane potential. Complex V uses the proton gradient to generate ATP. Antimycin (AA) inhibits Complex III and oligomycin (oligo) inhibits the ATP synthesis role of Complex V. (b) Graphical representation of Complex II subunit D, Complex III Cyt-c1 and Complex IV subunit 5A loss-of-function mutations. (c-f) Representative images of Control (c), Control + AA + oligo (d), Complex III Cyt-c1 (e), and Complex IV subunit 5A (f) mosaic ovaries 16-days post-clone induction incubated with tetramethylrhodamine, methyl ester (TMRM) to visualize the mitochondrial membrane potential (ΔΨ; magenta) live. Mutant germ cells do not express GFP (blue) and are outlined in white-dashed lines. Scale bars represent 10 μm. (g-i) Frequency of 5-, 11- or 14-days after clone induction of Complex II subunit D (g), Complex III Cyt-c1 (h), and Complex IV subunit 5A (i) mosaic ovaries. Undifferentiated: germline cells in Region 1 including GSC, cystoblasts, 2-, 4- and 8-cell cysts. Differentiated: germline cells in Region 2. Number of germaria observed are given inside the bars. P-values were calculated using Fisher’s exact test. For exact genotypes see S2 Table. (TIF) [file pgen.1010610.s005.tif]

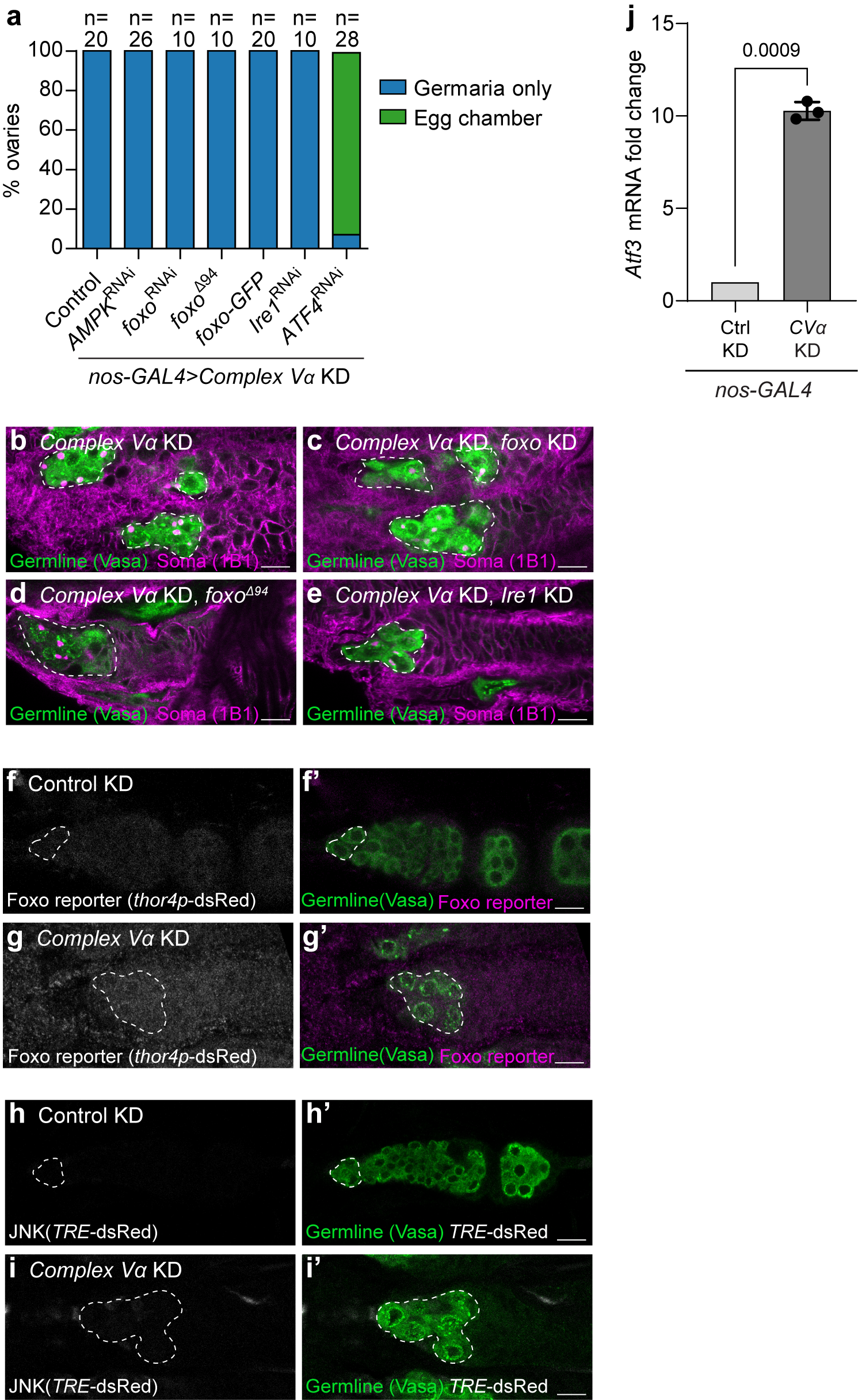

Supplement: S6 Fig — (a) Double knockdown of CV, and knockdown or overexpression of indicated mito-nuclear retrograde signalling genes. Number of ovaries analyzed is indicated at the above the bar. Ovaries were 1–7 days old. (b-e) Representative images of 2–3 day old CVα KD (b), CVα, foxo KD (c), CVα KD, foxoΔ94 mutant (d), and CVα, Ire1 KD (e) germaria. Images shown are representative of at least 100 germaria. (f, g) Representative images of 2–3 days old Control (f) and CVα KD (g) ovaries expressing FOXO activity reporter (magenta), thor4p-dsRed. (h, i) Representative images of 2–3 days old Control (h) and CVα KD (i) ovaries expressing JNK activity reporter (gray), TRE-dsRed. For (f-i) at least 30 ovarioles across five ovary pairs were analyzed. White-dashed line indicates the GSCs. For all confocal images, scale bars represent 10 μm. (j) Atf3 mRNA levels in Control (mCherry) KD and CVα KD ovaries expressing P35 to increase the number of germ cells. Data are the mean ± s.d. and statistical significance was calculated using unpaired t-test with Welch’s correction. All RNAi were driven by nos-GAL4. For exact genotypes see S2 Table. (TIF) [file pgen.1010610.s006.tif]

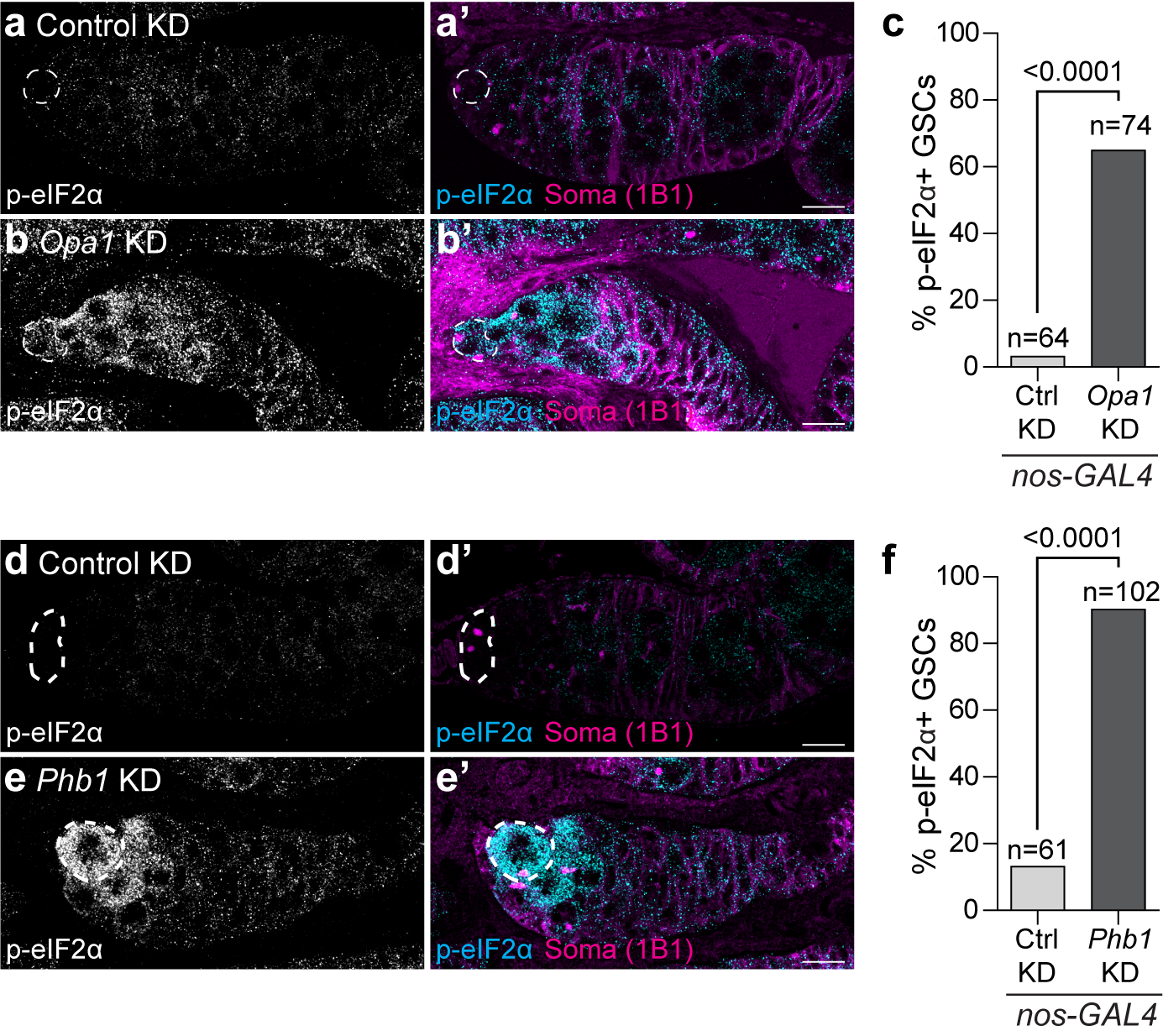

Supplement: S7 Fig — (a, b) Representative images of 2–3 day old Control (mCherry) (a), and Opa1 (b) KD germaria. (c) Frequency of GSCs with high phosphorylated eIF2α relative to surrounding somatic cells from (a, b). (d, e) Representative images of 2–3 day old Control (mCherry) (f) and Phb1 (e) KD germaria. (f) Frequency of GSCs with high phosphorylated eIF2α relative to surrounding somatic cells from (d, e). The number of GSC analyzed and P-value (Fisher’s exact test) are given above bars. For all confocal images, scale bars represent 10 μm and white-dashed lines demark the GSCs. All RNAi were driven by nos-GAL4. For exact genotypes see S2 Table. (TIF) [file pgen.1010610.s007.tif]

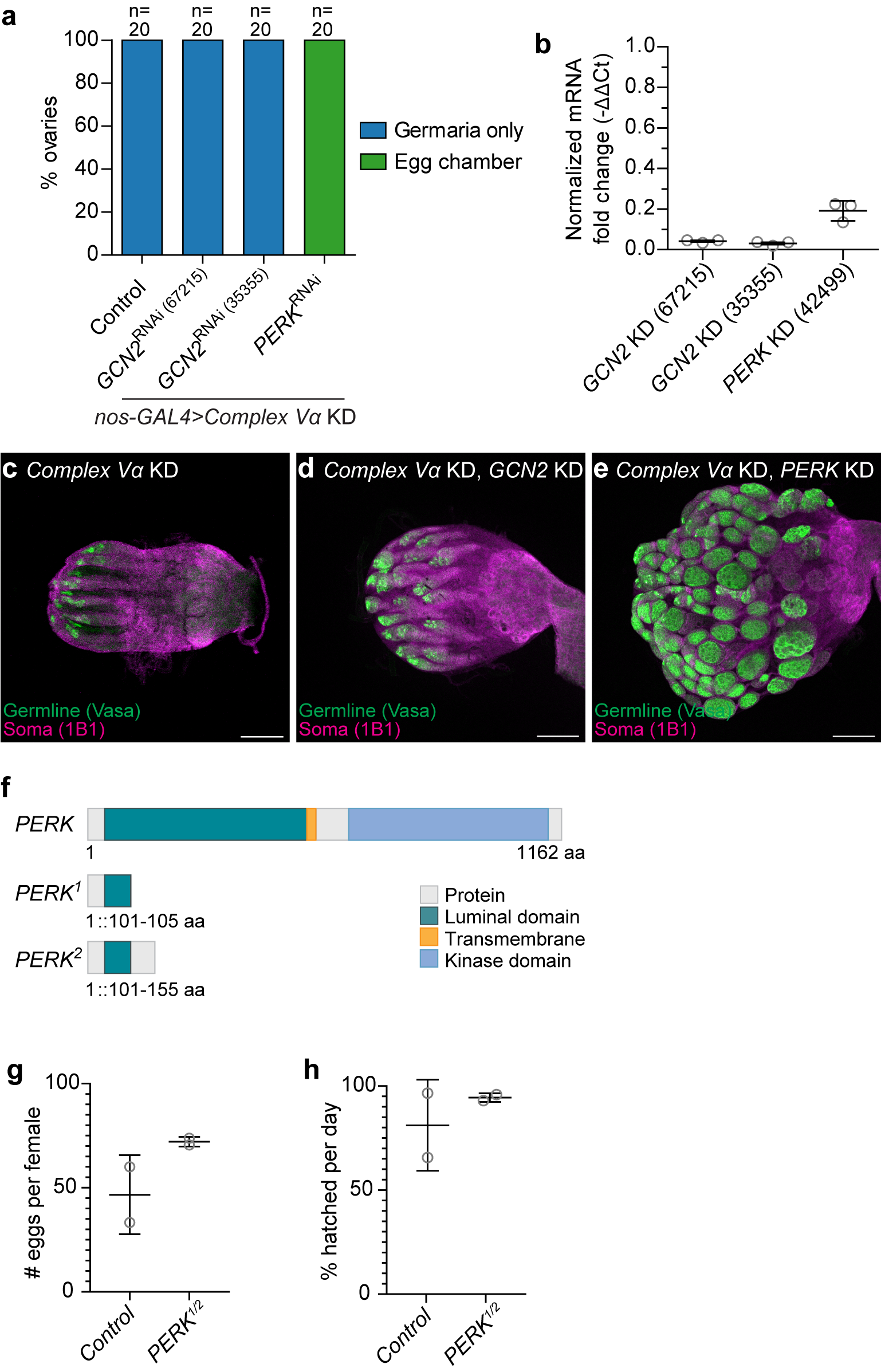

Supplement: S8 Fig — (a) Frequency of phenotype of Control (CVα KD only), GCN2 and PERK double CVα KDs driven by nos-GAL4. Number of ovaries analyzed are indicated at the top. Ovaries were 2–3 days old. (b) Knockdown efficacy of GCN2 and PERK RNAi lines used. RNAi were driven by maternal-tubulin-GAL4 and maternally deposited mRNA levels were assessed in less than 2 hour old embryos. RNA levels were normalized to Control (mCherry) RNAi. (c-e) Representative images of 2–3 days old CVα KD (c), CVα, GCN2 KD (d), and CVα, PERK KD (e) ovaries. Scale bars represent 100 μm. (f) PERK knockout alleles with 1 bp (PERK1) and 14 bp (PERK2) deletion are predicted to generate truncated and non-functional protein products. (g) Number of eggs laid per female of the Control (w1118) and PERK transheterozygous mutants (PERK1/PERK2). (h) Percent of hatched eggs laid by females in (h). Two replicates were performed for (g, h). For exact genotypes see S2 Table. (TIF) [file pgen.1010610.s008.tif]

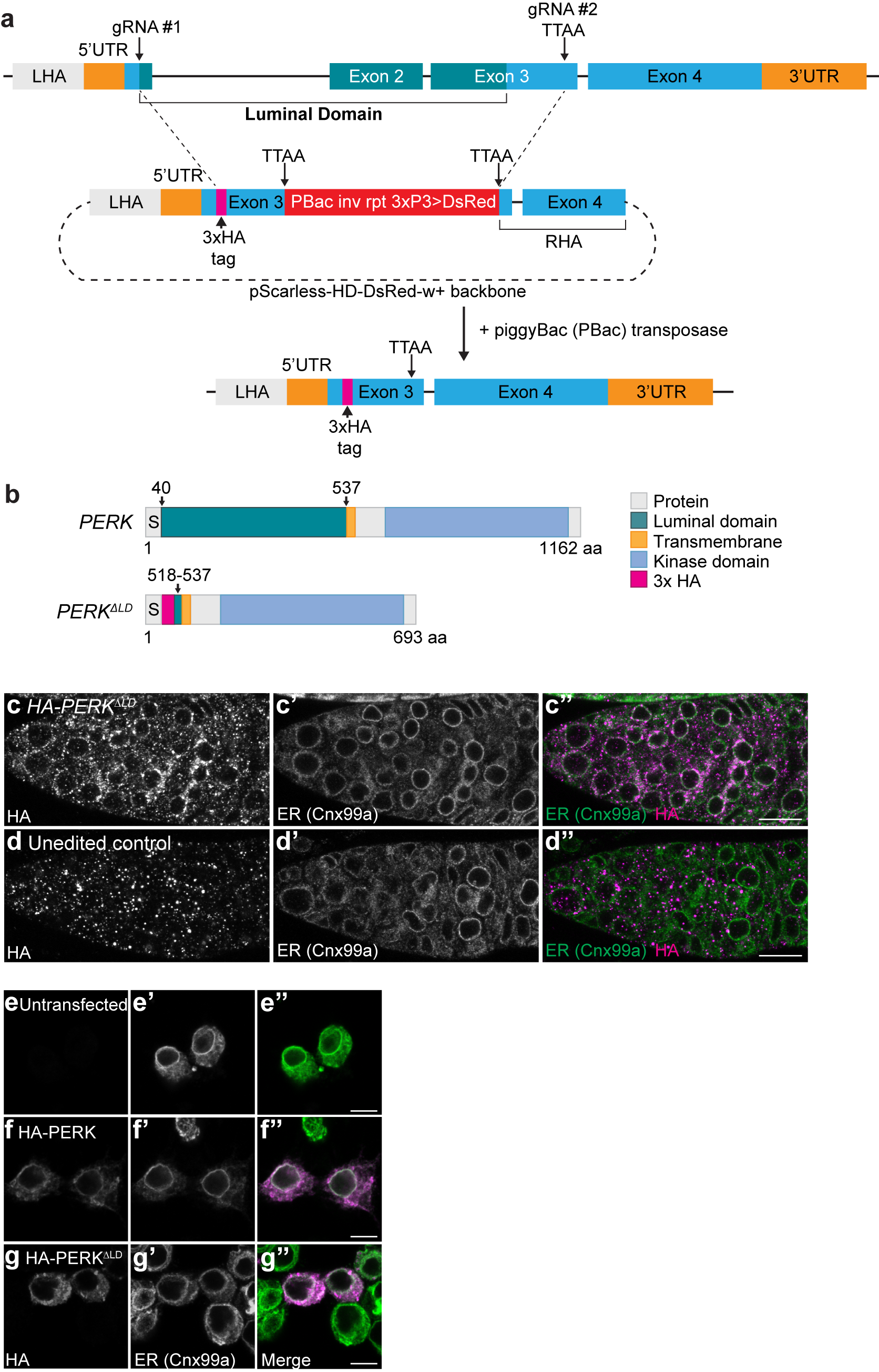

Supplement: S9 Fig — (a) Schematic of the generation of PERK lumenal domain deletion mutants using CRISPR/Cas9 and homologous recombination. Two guide RNAs were expressed flanking regions the PERK lumenal domain (exons 1–3). A plasmid containing the lumenal domain deletion and the piggyBac inverted repeat flanked 3xP3 driven dsRed was used to drive homologous recombination. After expression of piggyBac transposase, the dsRed region was excised scarlessly. (b) Graphical schematic of Drosophila wild-type and PERKΔLD domains. (c, d) Representative images of 2–3 day old homozygous PERKΔLD (c) and unedited control (w1118) (d) germaria. Scale bars represent 10 μm. (e-g) Representative images of untransfected S2R+ cells (e), and S2R+ transfected with HA-PERK (f) and HA-PERKΔLD (g). Scale bars represent 5 μm. For exact genotypes see S2 Table. (TIF) [file pgen.1010610.s009.tif]

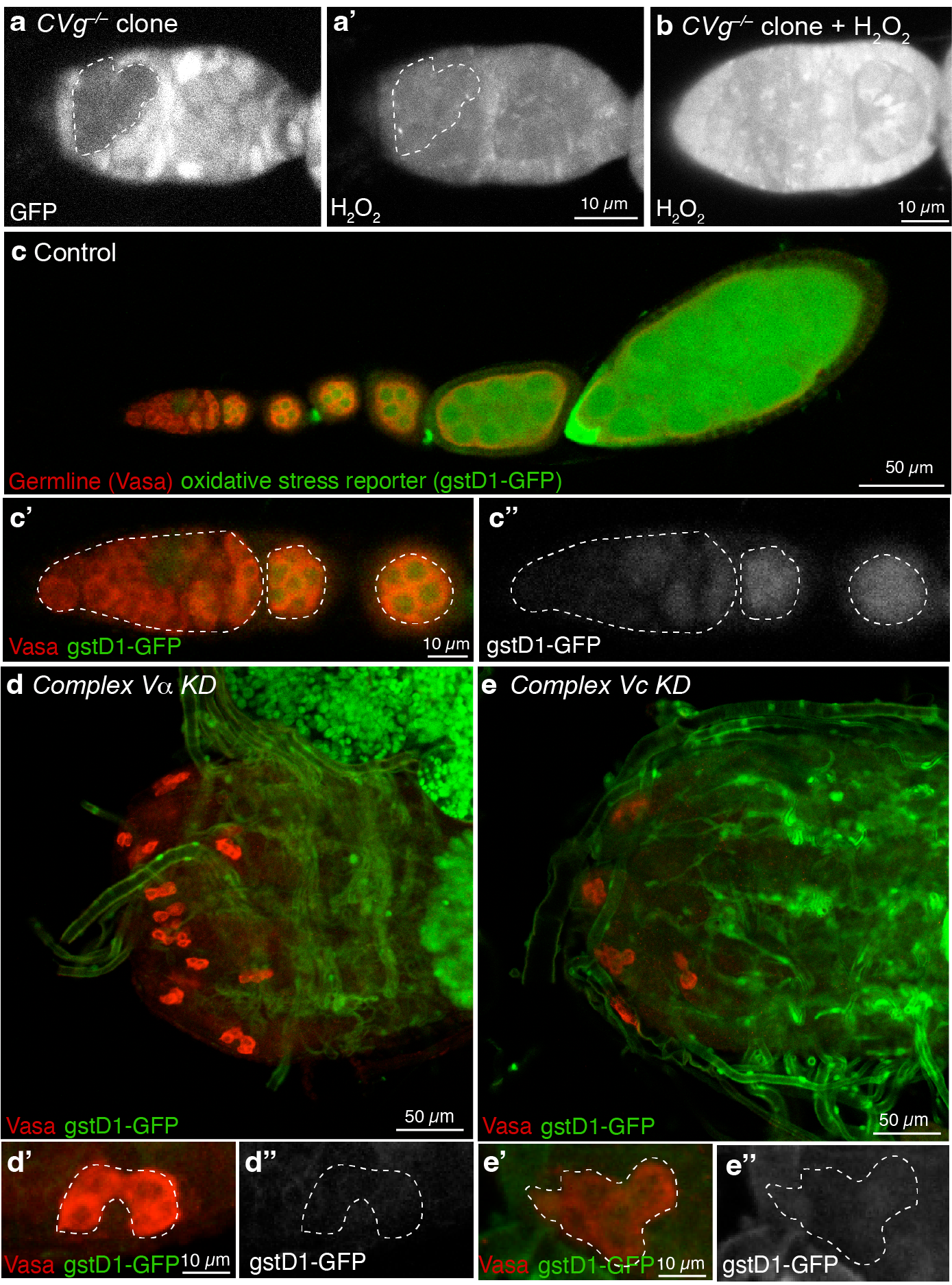

Supplement: S10 Fig — (a-b) No increase in hydrogen peroxide was observed in mutant CVg mosaic ovaries. Live representative image of CVg–/–mosaic ovaries 12-days post-clone induction incubated with the hydrogen peroxide sensor, peroxy orange-1 [110] (20 μM) in Schneider’s medium in the absence (a) or presence (b) of hydrogen peroxide (100 μM). GFP-negative cells marked by the white-dashed line represent CVg null mutant cells. (c-e) No increase in oxidative stress was observed CVa or CVc germline (white-dashed line) specific KD ovaries. Representative images of fixed ovaries Control (c), CVα (d) and CVc (e) germline KDs. Oxidative stress was assessed using the GstD1-GFP reporter [63]. Ovaries were fixed and stained with anti-Vasa to mark the germline and anti-GFP. For exact genotypes see S2 Table. (TIF) [file pgen.1010610.s010.tif]

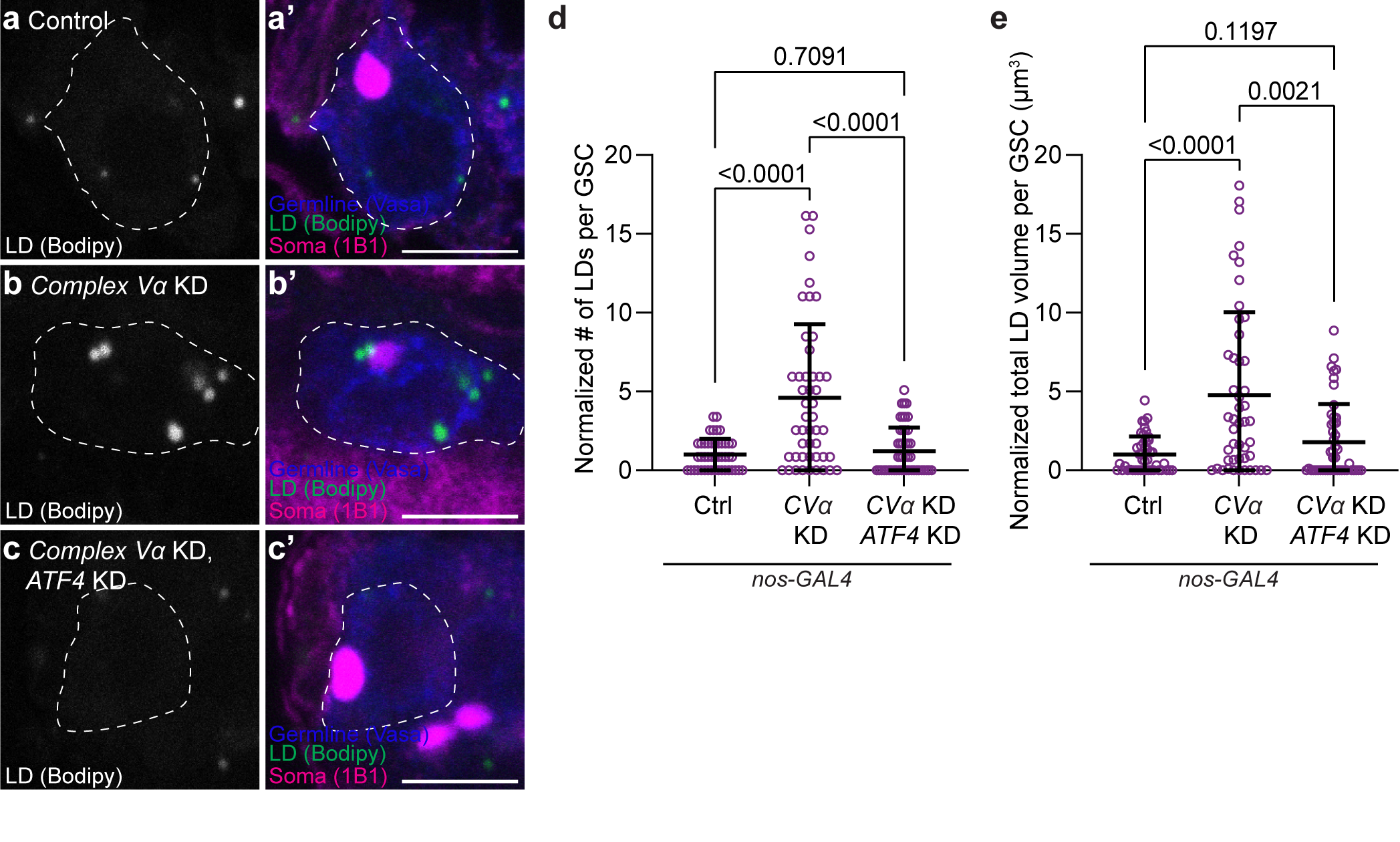

Supplement: S11 Fig — (a-c) Representative images of 2–3 day old Control (a), CVα KD (b), and CVα KD, ATF4 KD (c) GSCs (white-dashed line). BODIPY 493/503 marks lipid droplets. Scale bars represent 5 μm. (d) Quantification of number of lipid droplet per GSC normalized to mean of control for the indicated genotypes (n = 45 for Ctrl; n = 49 for CVα KD; and n = 47 for CVα KD, ATF4 KD). (e) Quantification of total lipid droplet volume per GSC normalized to mean of control for the indicated genotypes (n = 45 for Ctrl; n = 47 for CVα KD; and n = 47 for CVα KD, ATF4 KD). All RNAi were driven by nos-GAL4. For exact genotypes see S2 Table. (TIF) [file pgen.1010610.s011.tif]

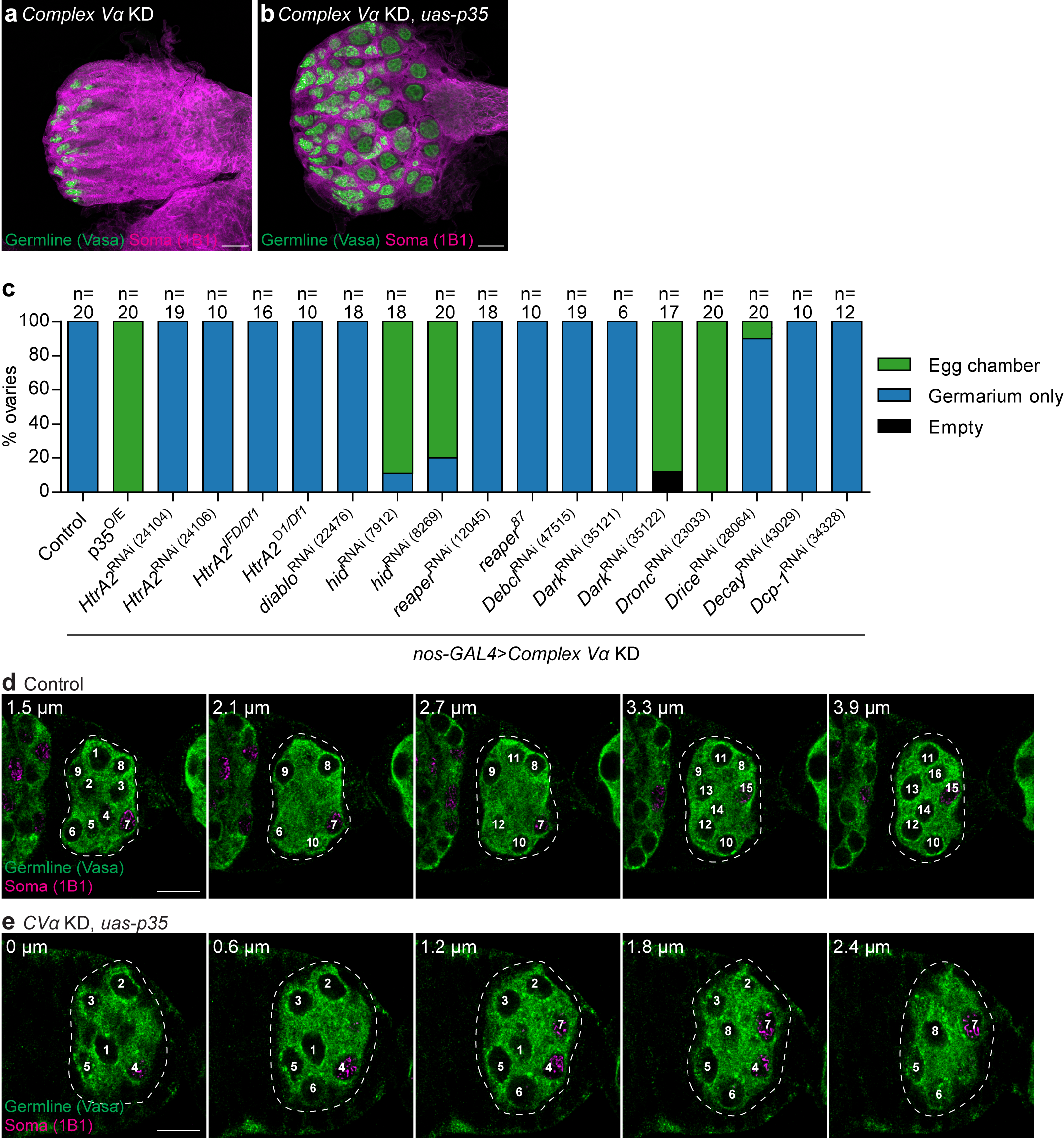

Supplement: S12 Fig — (a, b) Representative images of 1 day old CVα KD (a) and CVα KD, P35 overexpression (b) ovaries. Scale bars represent 50 μm. (c) Cell death suppressor screen in CVα KD background. Number of ovaries analyzed are indicated at the top. Ovaries were 1–4 days old. (e, f) Confocal image slices for (e) Fig 6D: Control and (f) Fig 6E: CVα KD with P35 overexpression germaria driven by nos-GAL4. White dashed lines outline the Region 3 egg chamber and numbers indicate cyst nuclei. Scale bars represent 5 μm. For exact genotypes see S2 Table. (TIF) [file pgen.1010610.s012.tif]

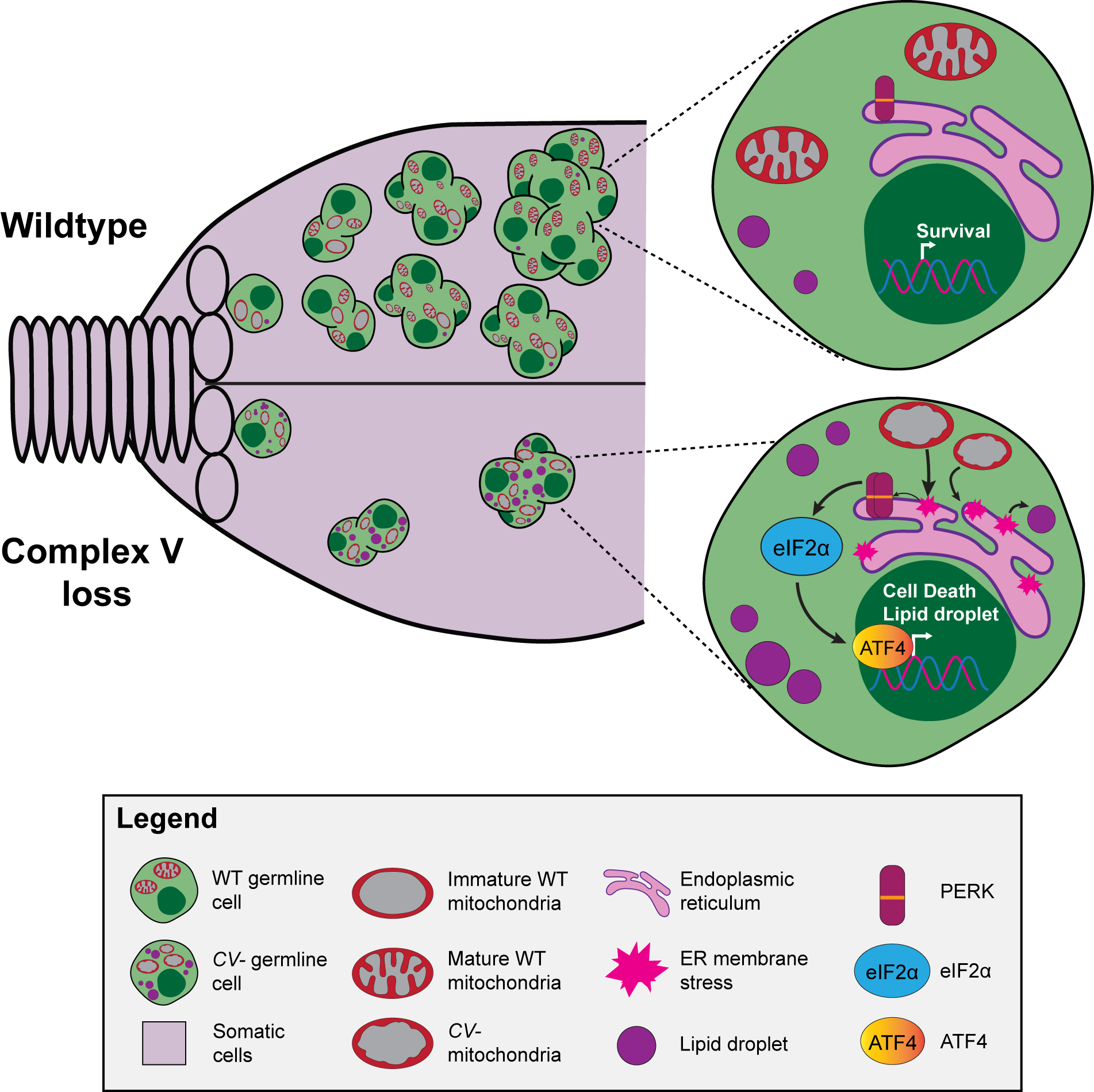

Supplement: S13 Fig — Schematic showing that the inability to remodel the inner mitochondrial membrane induces ER lipid membrane stress and cell death. ER bilayer stress is induced upon the loss of Complex V and cristae formation. This stress is detected by PERK which then activates the ISR through the phosphorylation of eIF2α and induction of ATF4. Increased ISR activity increases the formation of lipid droplets potentially to alleviate ER bilayer stress. (TIF) [file pgen.1010610.s013.tif]
